# The effects of radiofrequency electromagnetic fields exposure on human self-reported symptoms: A protocol for a systematic review of human experimental studies

**DOI:** 10.1016/j.envint.2021.106953

**Published:** 2022-01

**Authors:** Xavier Bosch-Capblanch, Ekpereonne Esu, Stefan Dongus, Chioma Moses Oringanje, Hamed Jalilian, John Eyers, Gunnhild Oftedal, Martin Meremikwu, Martin Röösli

**Affiliations:** a Swiss Tropical and Public Health Institute, Socinstrasse 57, CH-4051 Basel, Switzerland; b University of Basel, Petersplatz 1, CH-4003 Basel, Switzerland; c Department of Public Health, College of Medical Sciences, University of Calabar, Calabar, Nigeria; d Department of Biology, College of Art & Sciences, Xavier University, Cincinnati, OH 45247, United States; e Department of Occupational Health Engineering, Research Center for Environmental Pollutants, Faculty of Health, Qom University of Medical Sciences, Qom, Iran; f Independent Consultant & Senior Research Fellow, 3ie, c/o LIDC, 20 Bloomsbury Square, London WC1A 2NS, UK; g Department of Electronic Systems, Norwegian University of Science and Technology – NTNU, Trondheim, Norway; h Faculty of Medicine, College of Medical Sciences, University of Calabar, Calabar, Nigeria

**Keywords:** Radiofrequency electromagnetic fields, Electromagnetic hypersensitivity (EHS), Idiopathic environmental intolerance attributed to electromagnetic fields (IEI-EMF), Symptoms, Well-being, Sleep, Headache, Mobile phone

## Abstract

**Background:**

The technological applications of radiofrequency electromagnetic fields (RF-EMF) have been steadily increasing since the 1950s across multiple sectors exposing large proportions of the population. This fact has raised concerns related to the potential consequences to people’s health. The World Health Organization (WHO) is assessing the potential health effects of exposure to RF-EMF and has carried out an international survey amongst experts, who have identified six priority topics to be further addressed through systematic reviews, whereof the effects on symptoms is one of them. We report here the systematic review protocol of experimental studies in humans assessing the effects of RF-EMF on symptoms.

**Objective:**

Our objectives are to assess the effects of exposure to electromagnetic fields (compared to no or lower exposure levels) on symptoms in human subjects. We will also assess the accuracy of perception of presence of exposure in volunteers with and without idiopathic environmental intolerance attributed to electromagnetic fields (IEI-EMF).

**Eligibility criteria:**

We will search relevant literature sources (e.g. the Web of Science, Medline, Embase, Epistemonikos) for randomized trials (comparing at least two arms) and randomised crossover trials of RF-EMF exposure that have assessed the effects on symptoms. We will also include studies that have measured the accuracy of the perception of the presence or absence of exposure. We will include studies in any language.

**Study appraisal and synthesis:**

Studies will be assessed against inclusion criteria by two independent reviewers. Data on study characteristics, participants, exposure, comparators and effects will be extracted using a specific template for this review, by two independent reviewers. Discrepancies will be solved by consensus. Risk of bias (ROB) will be assessed using the ROB Rating Tool for Human and Animal Studies and the level of confidence in the evidence of the exposure-outcome relations will be assessed using the GRADE approach. For the perception studies, we will use adapted versions of the ROB tool and GRADE assessment. Where appropriate, data will be combined using meta-analytical techniques.

## Highlights

• There is public concern about the potential health effects of exposure to EMF.
• There is no updated synthesis of research evidence produced in experimental studies.
• We are considering the effects on symptoms.
• This evidence, together with evidence from other research syntheses, will inform future World Health Organization recommendations.

## 1 Introduction

### 1.1 Background and rationale

The technological applications of radiofrequency electromagnetic fields (RF-EMF; frequencies 100 kHz to 300 GHz) have been steadily increasing since the 1950s. RF-EMF are used in medicine (e.g. magnetic resonance imaging, diathermy, radiofrequency ablation), industry (e.g. heating and welding), domestic appliances (e.g. baby monitor, WiFi), security and navigation (e.g. radar and RFID) and especially in telecommunications (e.g. radio and TV broadcasting, mobile telephony). These developments mean that large parts of the global population are now exposed to RF-EMF and more will be exposed in the future. Concern has been raised regarding public health consequences from RF-EMF and it is therefore crucial to perform a health risk assessment to support decision-makers and to inform the general public.

The World Health Organization (WHO) has an ongoing project to assess potential health effects of exposure to RF-EMF in the general and working population, including patients. To prioritize potential adverse health outcomes from exposure to these fields, WHO conducted a broad international survey amongst RF experts in 2018 (Verbeek et al., 2021). Six major topics were identified (i.e. cancer, adverse reproductive outcomes, cognitive impairment, symptoms, oxidative stress, and heat-related effects) for which WHO has commissioned systematic reviews to analyse and synthesize the available evidence. In the current paper, we present the protocol for a systematic review on the effects of exposure to RF fields on symptoms evaluated in human experimental studies. In parallel, another protocol is developed for a systematic review the effects of the RF-EMF on symptoms evaluated in human observational studies (Röösli et al., 2021).

### 1.2 Description of the exposure

RF-EMF are defined as fields with frequencies from 100 kHz to 300 GHz. Such fields are generated by a large number of equipment both in the living environment and in workplaces. For these sources, a basic distinction is made between devices operating close to the body, resulting in a near-field exposure situation where RF-EMF is coupling to the body, and sources operating far away from the body, which produce a whole-body exposure from a quasi-homogeneous field (Lauer et al., 2013). The differentiation between near and far-field depends on several factors including the dimension of the transmitting antennas. Roughly, a far-field condition is obtained if the distance between transmitter and receiver is larger than a wavelength (ICNIRP, 2020). Typical near-field sources are mobile phones, Digital Enhanced Cordless Phone (DECT), computers, laptops and tablets. Typical far-field sources include radio- and television masts, mobile phone base stations, DECT base stations, Wireless Local Area Network (WLAN, WIFI) access points or mobile phone used at some distance. Many other RF-EMF sources are present in the living environment (e.g. baby monitors, smart meters, avalanche rescue beacons, remote control devices, antitheft devices), in occupational settings (RF polyvinyl chloride welding machines, plasma etching, radar systems) and in medicine (e.g. diathermy, magnet resonance imaging, cardiac pacemakers) (Hareuveny et al., 2015 Jan 30, Mantiply et al., 1997, Vila et al., 2016).

The main variables influencing the interaction of RF-EMF with the human body are the signal frequency (the higher the frequency, the lower the penetration depth), the exposure intensity (defined as the strength of the incident electric and magnetic fields or the incident power density), the exposure duration, the polarization of the field, the modulation of the signal and the dielectric characteristics of absorbing tissues. In most cases, the Specific Absorption Rate (SAR, in W/kg tissue weight) is the exposure measure of interest and, if multiplied by the exposure time, it represents the whole body-absorbed RF-EMF or the tissue specific energy dose. For local exposure at frequencies above 6 GHz, absorbed power density (S_ab_) is the relevant metric because the absorption mainly takes place superficially (ICNIRP, 2020). The SAR and S_ab_ depend on the frequency range of the signal, the field strength and the physiological characteristics of the absorbing tissue. For some experimental studies only external EMF exposure metrics are provided such as electric fields (V/m) or power density (W/m^2^). From this, SAR can be estimated using appropriate dosimetry for near-field or far-field conditions (ICNIRP, 2020).

### 1.3 Description of the health outcomes

The health outcomes to report in this review include (1) various symptoms in humans and (2) ability for humans to perceive the presence of RF-EMF.

Some people report several types of non-specific symptoms such as headache or sleep disturbances, which they relate to exposure to RF-EMF. Due to similarities to other forms of idiopathic environmental intolerance (IEI), such as multiple chemical sensitivity, this condition is referred to as IEI attributed to EMF (IEI-EMF) (WHO 2005), although according to a systematic review identifying IEI criteria, the most frequently used descriptive term was “hypersensitive to EMF” (Baliatsas et al., 2012b). The types of reported symptoms vary between individuals. The most commonly reported symptoms are headaches, sleep disturbances and tinnitus, among many others (Baliatsas et al., 2012a, Eltiti et al., 2007a, Eltiti et al., 2007b, Hillert et al., 1999 Nov, Oftedal et al., 2000 May, Röösli et al., 2004). To date, cluster analyses have not identified specific symptom clusters related to specific EMF exposure sources or to EMF exposure in general (Röösli et al., 2004) and the pattern of symptoms does not seem to be part of any recognized syndrome (ANSES, 2018).

To date, the definition of the condition has no objective diagnostic criteria and relies on self-attribution of symptoms to EMF (WHO, 2005) in the absence of other causes and a temporal relation between exposure and the appearance of symptoms (Baliatsas et al., 2012b, Dieudonné, 2020 May 6). Prevalence of IEI-EMF was found to substantially vary between years and countries; for example: 1.5% in Sweden (Hillert et al., 2002), 3.2% in California (Levallois et al., 2002), 3.5% in Austria (Schröttner and Leitgeb, 2008) and in The Netherlands (Baliatsas et al., 2015), 5% in Switzerland (Schreier et al., 2006a, Schreier et al., 2006b), about 10% in Germany (Blettner et al., 2009), 13% in Taiwan in 2007 (Meg Tseng et al., 2011) and 4 % in Taiwan five years later (Huang et al., 2018).

A substantial proportion of IEI-EMF individuals report to react within minutes to EMF exposure (Röösli et al., 2004). However, various studies indicate a nocebo effect (Munnangi et al., 2020) as a potential explanation of the symptoms in contrast to or in addition to physical exposure (e.g. Eltiti et al., 2007a, Oftedal et al., 2007, Rubin et al., 2010, Wallace et al., 2012). The term “nocebo effect,” derived from the Latin nocere meaning “harm,” is commonly used when a placebo causes an unfavourable outcome and means that symptoms may be provoked by the belief of a harmful exposure. Indications for nocebo reaction come from studies that applied an open provocation, i.e. study participants were informed about the presence and absence of RF-EMF exposure during the experiment, in addition to a blinded provocation (Eltiti et al., 2007a, Wallace et al., 2012) or as a recruitment instrument (Oftedal et al., 2007, van Moorselaar et al., 2017). Acknowledging the complexity of mechanisms that could explain a nocebo effect, proper blinding is needed to reduce the risk for bias when exploring whether the physical presence of the EMF fields causes acute symptoms. It is also conceivable that RF-EMF triggers non-specific symptoms in people who do not perceive themselves as having the features of IEI-EMF (Baliatsas et al., 2012a). Therefore, studies carried out in the general population are also important to assess the effects of RF-EMF.

Assessing effects on symptoms in a controlled experiment (e.g. testing effects of exposure and no exposure by using cross-over or parallel designs) is the most direct way of exploring the role of the RF-EMF exposure for persons with IEI-EMF, since the condition is characterised by the connection between symptoms and exposure. Given that many of those attributing symptoms to EMF exposure report that they can perceive the RF-EMF during and shortly after exposures (e.g. Röösli et al., 2004), it is also useful to consider studies that report the ability to perceive or sense RF-EMF ( called EMF perceptions, from now on) when reviewing the literature. In experimental provocation studies, people with IEI-EMF report that they are exposed to RF-EMF more often than people without this condition, both when being actually exposed to RF-EMF and when being sham-exposed (i.e. no actual exposure) (e.g. Nam et al., 2009). Thus, it is important to evaluate whether the testing approaches consider comparisons between different types of populations. It is also important to clarify whether individuals with IEI-EMF are able to perceive RF-EMF more accurately than individuals without IEI-EMF. If they are not, the perception is not related to their condition of IEI-EMF and this would not support the existence of a physical interaction. On the other hand, a higher ability to perceive the exposures compared to people without IEI-EMF, suggests a physical interaction mechanism irrespectively of how this is clinically expressed.

### 1.4 Rationale for the systematic review

Several literature reviews have been carried out to assess whether RF-EMF levels below regulatory limits may cause symptoms or may be perceived by volunteers with and without IEI-EMF. No evidence for an effect of the exposure has been found in these reviews that included population-based observational studies (Baliatsas et al., 2012a, Röösli, 2008, Röösli et al., 2010) or experimental studies (Rubin et al., 2010, Röösli, 2008, Schmiedchen et al., 2019 Oct 22). Randomized human experimental studies are most appropriate to investigate the triggering of symptoms within a short time after exposure and thus, an updated review on human experimental studies is justified. Short-term exposure refers to a maximum of a few hours in human laboratory trials and a maximum of a few days in experimental field studies. Long term studies are required to assess the potential of delayed, long-term effect, which are evaluated in another systematic review(Röösli et al 2021).

## 2 Objectives

Our aim is to assess the evidence on the relation between short term exposure to RF-EMF and acute symptoms, attributable to a physical mechanism related to the exposure, beyond a nocebo effect; and whether the presence of RF-EMF exposure below the levels of the ICNIRP guidelines can be perceived.

In particular, the PECO question are (Morgan 2018):

- *in volunteers with IEI-EMF and without IEI-EMF (P), is exposure to RF-EMF (E), as compared to no or lower exposure levels (C), related to immediate effects on symptoms (O)?*
- *in volunteers with IEI-EMF and without IEI-EMF (P), are different exposure levels to RF-EMF (E, C) (*e.g. *intensity, duration) related to the intensity of self-reported symptoms (O)?*

We will also compare effect sizes between studies with blinded and non-blinded participants for the exposure situation and for participants with and without IEI-EMF.

We define immediate effects as outcomes that have occurred during or within 24 h after exposure as recorded. We will not attempt to estimate potential delayed, long-term effects.

## 3 Methods

The review will be carried out following the recommendations in the WHO Handbook for Guideline Development (WHO 2014) and COSTER (Recommendations for the conduct of systematic reviews in toxicology and environmental health research) (Whaley et al., 2020). It will be reported in accordance with the Preferred Reporting Items for Systematic Reviews and Meta-Analyses (PRISMA) guidelines (Page et al., 2020).

### 3.1 Eligibility criteria

#### 3.1.1 Type of populations

We will include people of any age, gender, occupation or socio-economic condition, including pregnant women and people with any health status, with and without IEI-EMF. If no studies are found for specific types of population, we will state it in the results section.

#### 3.1.2 Type of exposure and exposure assessment

We will include all studies that have applied electric, magnetic or electromagnetic fields in the frequency range of 100 kHz to 300 GHz. We will include studies applying near-field exposures (e.g. by mimicking a mobile or cordless phone call) or far-field exposures (e.g. applying a nearly homogeneous field to the whole body).

##### 3.1.2.1 Inclusion criteria

We will include studies both with participants that are blind to and also aware of the exposure situation. We will base our overall estimates only on the studies that have blinded the participants to the exposure situation because such a design reduces potential nocebo effects. We will use other studies with non-blinded exposure conditions to assess the effect of blinding and to evaluate nocebo effects. We will not attempt to establish the underlying mechanisms that would explain observed nocebo effects.

For any study to be eligible, at least one of the exposure descriptions listed below needs to be reported:

A) body or organ internal exposure metrics measured or calculated for the particular conditions of the experiment as follows:
• SAR [expressed in W/kg or equivalent units]
• SA [Specific energy Absorption, expressed in J/kg or equivalent units]
• induced electric field strength, [expressed in V/m or equivalent units]
• internal magnetic field strength [expressed in H/m or equivalent units]

(for exposure applied as pure or predominantly magnetic fields in the lower frequency range, the external magnetic field strength at sample position is considered a sufficient surrogate for the tissue internal magnetic field as long as the penetration depth is high compared to the sample dimension);

B) exposure metrics describing superficial body absorption at frequencies above 6 GHz measured or calculated for the conditions of the experiment as follows:
• absorbed power flux density [expressed in W/m^2^ or equivalent units]
• absorbed energy density [expressed in J/m^2^ or equivalent units]
C) body external exposure metrics
• incident electric field strength [V/m]
• incident magnetic field strength [mA/m]
• incident power flux density, [expressed in mW/m^2^]
• incident energy density [expressed in J/m^2^].

We will only include studies reporting external metrics as under C if (i) either of these exposure metrics was measured or calculated at the location of the exposed body in the approximate far-field of the field source, and (ii) the exposure level is at least a factor of 10 (power flux density and energy density) or √10 (field strength) above background level. In the case where no specific background exposure level in the laboratory is reported in the study, we will assume a value of 0.25 mW/m^2^ (corresponding to 0.3 V/m and 0.9 mA/m, respectively) as the background exposure level (Jalilian et al., 2019). This results in an inclusion threshold of PD = 2.5 mW/m^2^, E = 1 V/m, or H = 2.7 mA/m.

D) Mobile phones or other RF-generating devices as source of exposure without reporting of metrics under A, B or C. We will consider these studies separately because there is variation and/or uncertainty in exposure levels.
• We will include studies that applied exposure with an output power controlled by hardware or software, provided that the output power and the distance to the sample are reported, enabling inference of the exposure.
• Studies using a mobile phone with an active call operated close to the body will only be included if the active call was maintained throughout the experiment and measures were described to prevent discontinuous transmission mode (DTX) from being activated and the comparison was a similar phone switched off.

##### 3.1.2.2 Exclusion criteria

We will exclude studies that have applied exposure signals with >10% of the total signal energy outside the considered frequency range 100 kHz – 300 GHz (e.g. pulsed fields, non-sinusoidal fields with dominant frequencies <100 kHz). We will not exclude studies on the grounds of the intensity or duration of exposure.

#### 3.1.3 Type of comparators

We will include studies with at least one active exposure condition and one control condition that may be either sham exposure (i.e. no exposure beyond background exposure) or an RF-EMF exposure at a lower level. The way exposure level is reported in exposed groups or arms should be the same or it should be possible to standardise them.

#### 3.1.4 Types of outcomes

*Symptoms*

A symptom is an effect noticed and experienced by the person who may have a condition. Outcomes have been estimated in several ways, including composite scale measurements of well-being, such as the von Zerssen score (von Zerssen 1976) or questionnaires that would identify the key symptoms associated with IEI-EMF (Eltiti et al., 2007a, Eltiti et al., 2007b). We will include any symptoms as inquired in eligible studies. We consider, symptoms commonly reported, such as headache, sleep quality measures and composite symptom scores as the main outcomes of this review. We will also consider other symptoms that affect health-related quality of life (e.g. fatigue, exhaustion, nervousness). The nature of these outcomes means that they will be self-reported and they may not necessarily reflect a disease. The severity of symptoms may be expressed as a score or binary variable (presence/absence of symptom).

This systematic review belongs to a series of reviews commissioned by WHO and is focusing on symptoms. Therefore, we will not consider in this review physiological outcomes, such as those measured with physical devices (e.g. laboratory tests, blood pressure measurements, imaging; not even if those findings are self-reported by participants (Rubin et al., 2011) because these findings cannot be considered as reliably when self-reported.

*Perception of exposure*

To assess the accuracy of RF-EMF exposure perception, we will include studies that have tested the ability to perceive RF-EMF as a self-report of perception. We will include any such studies regardless of the methods and/or scales used to report perceptions and whether that was the primary or secondary outcome.

#### 3.1.5 Types of studies

##### 3.1.5.1 Inclusion criteria

We will include trials with RF-EMF exposure, conducted either in laboratories or at any other locations, such as in homes or workplaces:

• randomized trials comparing at least two arms exposed to different intensities of EMF (i.e. parallel group trials);
• randomised crossover trials in which each participant receives all exposure (or alternative exposure) conditions and is randomly allocated to the sequence of those conditions (i.e. crossover trial).

##### 3.1.5.2 Exclusion criteria

We will exclude studies reporting exposure effects on implants such as pacemakers or cochlear implants (Sorri et al., 2006). This interaction is well understood and avoided by proper electromagnetic compatibility testing of implants and is thus not considered in this review.

##### 3.1.5.3 Years considered

All publication years will be considered.

##### 3.1.5.4 Publication language

No language restriction will be applied. Articles in languages other than the ones spoken by the reviewers (English, German, Spanish, Catalan, French and Portuguese) will be presented to collaborators in the network of authors’ institutions proficient in those languages to assess inclusion and perform data extraction. However, considering that title and abstract of non-English articles published in peer-reviewed journals are in English, only English terms will be used to search the publication databases.

##### 3.1.5.5 Publication types

Only peer-reviewed published studies will be considered. Based on findings from related systematic reviews we consider that evidence uniquely reported in grey literature will not significantly change the findings from peer-reviewed published studies, and will not be considered.

#### 3.1.6 Types of effect measures

Symptoms

- For dichotomous outcomes (i.e. symptom yes/no) we will report rate ratios based on the number of persons that report one or more symptom in the intervention and control arms.
- For discrete or continuous outcomes (i.e. scales of severity of symptoms) we will report mean differences. If different measurement tools are used for the same category of symptoms, we will use Standardised Mean Differences.
- If available data does not allow these calculations, we will report the effect measures in the most detailed possible way, according to the data available in the studies.

Perception

- For dichotomous ratings (presence/absence of field), we will extract the number of correct ratings, as well as false-positive and false-negative recordings during or up to 24 h after exposure. For each study and each exposure level, we will calculate the sensitivity, specificity and diagnostic odds ratio (dOR).
- If perception is expressed with a discrete or continuous variable (e.g. perception of the ‘intensity’ of exposure) we will estimate mean differences.

We will estimate 95% confidence intervals for all effect measures. Note that confidence intervals do not measure the likelihood that a reported symptom is actually accurately reported, but it rather estimates the precision of the point estimates across subjects and studies. Since we do not have a gold standard for symptoms, we will be unable to assess sensitivity or specificity of reported symptoms.

If data necessary for analysis are missing from the articles, we will ask the authors to provide them. If this does not succeed, we will calculate the missing data from other data that are reported. If only effect sizes are available, we will use these as reported in the studies and their 95% confidence intervals.

### 3.2 Information source and search strategy

Eligible studies will be identified by literature searches (see Appendix 1) in the following databases: Medline, Web of Science, PsycInfo, Cochrane Library, Epistemonikos and Embase. Each database strategy will be tailored to its specific search syntax. The strategy will use two study design filters – observational (Röosli et al., 2021) and experimental studies for this review – as outlined above in the inclusion criteria of study types. We will also consult the EMF-Portal, a dedicated database of the scientific literature on the health effects of exposure to electromagnetic fields (https://www.emf-portal.org/en). Searches will be supplemented by checks of the reference lists of previous systematic reviews. We will use Endnote to manage references.

### 3.3 Study selection

After the retrieval of references, we will identify and discard duplicates. First, the relevance of identified papers will be checked on the basis of titles and abstracts, to discard animal studies or studies obviously out of the scope of this review. The full text of relevant references will be obtained to assess whether they fulfil all the inclusion criteria, independently by the two reviewers. If there are disagreements on the decisions made, these will be resolved by consensus between the two reviewers, and with a third reviewer, if necessary. If a certain study is described in more than one article with complementary results, we will use the results as reported in all available articles and refer to it as a single study. If a single document reports several studies, we will individually consider each one of the studies separately. This will result in a list of included studies. Excluded studies, and reasons for exclusion, will be also listed in the list of excluded studies. We will document the selection process in a study flow diagram according to the PRISMA reporting guidelines (Liberati et al., 2009, Moher et al., 2009).

### 3.4 Data extraction

Two reviewers will independently extract and record the relevant data of each eligible study. If any of the authors of the review is also an author of an included study, we will make sure that this author will not extract data from their own study and will not judge the RoB. Discrepancies will be solved by checking the source. If disagreement occurs between the reviewers, this will be resolved by discussion; if no consensus can be reached a third reviewer will be involved. Data will be extracted and described in a table of included studies:

- Citation
- Study design
- Type of environment (e.g. laboratory, home)
- Participants information (e.g. age, gender, IEI-EMF status with types of RF-EMF exposure that are experienced to cause the symptoms if applicable, education level, socio-economic and health conditions)
- Number of participants with and without IEI-EMF, enrolled and analysed
- Exposure details (e.g. source, frequency, modulation and duty circle, time pattern of exposure (on–off periods for intermittent exposure), part of body exposed, distance to source
- Time-average SAR(s), time-average exposure level(s) (incident electric/magnetic field strength or incident power density) or, if exposure levels are not provided, time-average output power(s)
- Adaptation period, exposure duration, and time between exposure conditions
- Background exposure level
- Co-exposures (intensity/dose and timing relative to the RF-EMF exposure)
- Other exposures and factors that might affect the symptoms
- Confounders
- Outcomes assessed: type and specification of outcomes, times of recording, methods of measurement
- Effects of the exposure as reported by studies
  o *Symptoms*: number of participants with each reported symptom and well-being level recorded before, during and after exposure (or changes from baseline if only changes are provided) for the exposure and control conditions; or means (or medians) and a measure of dispersion, if applicable;
  o Well-being has been described in terms of anxiety, tension, arousal, relaxation, discomfort, fatigue, depression and confusion (Eltiti et al., 2007a, Furubayashi et al., 2009 Feb). We will also report on well-being issues as reported by authors.
  o *Perception*: number of volunteers that perceived exposure or difference in exposures correctly and incorrectly (both false-positives and false-negatives) perceived.
- Effect sizes as reported in the studies and their 95% confidence intervals
- Data by groups or arms (e.g. number of subjects, number of events, scales or values of continuous variables, measures of dispersion and other relevant data required to consider re-analyses or meta-analyses).

We will also extract items to estimate the Risk of Bias (ROB, see below). For missing data in articles published in the last ten years, we will contact authors and ask for additional information. We will assume no symptoms before exposure if pre-exposure data for the symptoms are not available or if only the change from the pre-exposure level is provided.

### 3.5 ROB assessment

For evaluating the internal validity, we will conduct a ROB assessment using the “ROB Rating Tool for Human and Animal Studies” developed by the NTP Office of Health Assessment and Translation (OHAT) (NTP. OHAT, 2015, Rooney et al., 2014), which was adapted for the specific exposure and outcomes considered in this review. We only consider domains relevant to “Human Controlled Trials”. In the ROB form (Appendix 2), all instructions from the original OHAT document (NTP 2015) are typed in black. All adaptions to the form, which were informed by the review team and discussions with other WHO review teams and COSTER (Whaley et al., 2020) are typed in blue for easier recognition. Studies will be assessed across six domains with eight different questions, with detailed criteria elaborated for each domain in the ROB instructions (Appendix 2). The following issues are considered: randomisation of exposure level, allocation concealment, blinding, attrition level, exposure characterisation, outcome assessment and reporting, funding sources and other sources of bias.

One reviewer will extract and record the relevant features of each included study. A second reviewer will check the extracted study information against the accompanying article(s) for completeness and accuracy as a quality control measure. The ROB for each study will be conducted separately by both reviewers.

Using the instructions guide (Appendix 2), ROB will be assessed at outcome level and therefore studies reporting on several outcomes will have the corresponding ROB assessments. Following the OHAT ROB classification, ROB will be reported as definitely low ROB(++), probably low ROB(+), probably high ROB (- or not reported “NR”), or definitely high ROB (--) for each criteria. ROB may result into a false positive risk (i.e. overestimation of harmful effect), bias towards absence of an association (i.e. underestimation of harmful effect), false protective finding (i.e. favours beneficial effect) or unpredictable effect.

A challenge particularly in cross-over studies is confounding from carry-over as well as diurnal or day-to-day variation in health-related quality of life. To address these issues, we will take into account whether studies include a sufficiently long wash-out period between different exposures and whether authors have considered ‘period’ effects in the design or in the analytical stages of the studies including diurnal and day-to-day variation of the outcomes. We will assume the length of “wash-out” periods reported by authors to be optimal provided that (i) periods are not shorter than the study defined time lapse between exposure and symptoms and (ii) reported symptoms have disappeared. We will also take into account the selective first period reporting in the ROB tool.

*Key criteria for 3-Tier system*

As suggested by the OHAT handbook, we will apply a 3-Tier system for later synthesizing study findings when ROB vary across studies or across different analyses from the same study. The tier approach is based on the following three key criteria:

1. Can we be confident about the random and concealment of allocation of subjects (instead of “did the study design or analysis account for important confounding and modifying variables?", applied to observational studies).
2. Can we be confident in the exposure characterization?
3. Can we be confident in the outcome assessment?

We will produce heat maps for visualization of the ROB. Note that within the same study, the results of the tier approach may vary depending on the type of outcome, the type of exposure and the type of the exposure assessment method considered.

A Tier 1 study result must be rated as “definitely low” or “probably low” ROB for key criteria and have the rest of criterial as “definitely low” or “probably low” ROB. A Tier 3 study result must be rated as “definitely high” or “probably high” ROB for key elements AND have all other applicable items as “definitely high” or “probably high” ROB. A Tier 2 study result meets neither the criteria for 1st or 3rd Tiers (NTP 2019).

Funding source and disclosure of conflict of interest is not a specific domain in the OHAT ROB tool, but we will collect such information during data extraction. Funding source is recommended as a factor to consider when evaluating ROB of individual studies for selective reporting and then again for evaluating the body of evidence for publication bias. Funding source should be considered as a potential factor to explain apparent inconsistency within a body of evidence. Based on empirical evidence (Huss et al., 2007) we may consider bias in both directions, e.g. downplaying associations because of industry bias or highlighting associations to attract research funding, for instance in unfunded pilot studies.

#### 3.5.1 Publication bias assessment

If there are more than five studies, publication bias will be assessed using a funnel plot and applying Egger's test to the included studies.

### 3.6 Synthesis of the results

We will combine the effects of local and whole-body exposure from studies that are considered clinically and statistically sufficiently similar (see section on heterogeneity 3.6.1) in a random effects meta-analysis with STATA version SE 15. We will first pool the effect sizes of the highest exposure contrast per study.

If symptoms, well-being or perceptions have been recorded at different points in time, we will use the assessment that measured the largest severity or, if no severity reported, the assessment closest to the end of exposure, in the meta-analysis.

To assess relations between exposure doses and the effects on symptoms, we will carry out meta-regression analyses, where exposure will be regarded as a continuous variable. The following exposure measures will be applied for local and whole-body exposure:

*Local exposure with frequencies up to and including 6 GHz*

The exposure metric to be applied is the spatial peak SAR averaged over 10 g of any tissue (SAR_10g_). When maximum local SAR_1g_ for the outer layer of the head is provided, we will convert to SAR_10g_ for the same region, as follows (Hirata et al, 2006): SAR_10g_ = 0.6 *SAR_1g_.

*Local exposures with frequencies above 6 GHz*

The exposure metric to be applied is the surface area peak S_ab_ averaged over a square 4-cm^2^ surface area of the body.

*Whole-body exposure for all frequencies*

The exposure metric to be applied is the whole-body average SAR. Exposure quantifications using the incident electric field, magnetic field or the power density will therefore be converted into whole-body average SAR. We will do this by applying the correspondence between the ICNIRP (2020) basic restrictions (whole-body average SAR) and the reference levels (incident power density, incident electric field and incident magnetic field), and we will apply the values for general public where the basic restriction for whole-body average SAR is 0.08 W/kg. To estimate the whole-body average SAR (SAR_WBA_), the following formula will be used:

• SAR_WBA_ = 0.08 W/kg × P_i_,_study_/P_i_,_RL_ where P_i_,_study_ and P_i_,_RL_ are the incident power density of the study and the ICNIRP (2020) reference level for general public, respectively.
• SAR_WBA_ = 0.08 W/kg × (E_i_,_study_/E_i_,_RL_)^2^ where E_i_,_study_ and E_i_,_RL_ are the incident electric fields of the study and the ICNIRP (2020) reference level for general public, respectively.
• SAR_WBA_ = 0.08 W/kg × (H_i_,_study_/H_i_,_RL_)^2^ where H_i_,_study_ and H_i_,_RL_ are the incident magnetic fields of the study and the ICNIRP (2020) reference level for general public, respectively.

These formulas will be used for exposure frequencies above 300 MHz that are not k-polarised, because then the effect of polarization is only insignificant (Durney et al., 1986, Hirata et al., 2009, Kühn et al., 2009, Vermeeren et al., 2008). For exposures with k-polarisation or at frequencies below 300 MHz, i.e. closer to or at the whole-body resonance frequencies, a conversion to whole-body average SAR cannot be done due to too large uncertainties.

To assess the accuracy of the perception of exposure we will estimate chance corrected perception odds ratio taking into account expected correct ratings based on chance according to the underlying testing design as described in Röösli et al, 2008.

We will estimate the association between RF-EMF exposure and symptoms from studies where participants were blinded. To assess the effect of blinding we will compare results of studies that applied double-blinded, single-blinded and unblinded (open provocations) conditions using meta-regression. A significant difference of the effect sizes between these conditions indicates the relevance of blinding, which would suggest the presence of a nocebo effect.

In addition, to assess the nocebo effect, we will estimate the effects on symptoms in experimental studies where participants were in one case told that they would be exposed to RF-EMF and in another that they would not be exposed, while they were actually not exposed in any of those conditions. The difference in effect under these conditions would be a measure of the nocebo effect.

#### 3.6.1 Assessment of heterogeneity

We will consider as heterogeneous and do not combine the following studies: people with and without IEI-EMF; local, near field exposure below and above 6 GHz and whole-body exposure; blinded and unblinded studies.

We will consider as similar all symptoms; RCTs and cross-over trials; all times of outcomes measurements; and all perceptions of the presence of exposure.

We will use Stata to assess statistical heterogeneity by means of the I^2^ statistic. We will take the values of I^2^ of 25%, 50% and 75% as low, moderate and high degrees of heterogeneity respectively. We will also calculate Tau-square and calculate an 80% prediction interval.

#### 3.6.2 Sensitivity analysis

We will assess if results are sensitive to the inclusion of low-quality studies with a high ROB (tiers 2 and 3) by analysing separately studies with low ROB and then adding studies with high ROB.

#### 3.6.3 Subgroup analysis

There might be a modification of the effects depending on variation in PECO elements.

Therefore, we will conduct the following subgroup analyses:

- by symptom
- by gender: comparing the effects of studies with male and with female participants
- by IEI-status and health status (e.g. health and sick)
- by age groups: comparing the effects of studies with adults and children
- by types of signals; comparing the effects of studies with:
  o signals with low-frequency temporal characteristics like *Time-division multiple access* (TDMA) signals as for GSM;
  o signals with intermittent pattern of the order of milliseconds like in *Long-Term Evolution Time-Division Duplex* (LTE-TDD), also called *Time-Division LTE* (TD-LTE);
  o versus any other types of signals.
- by duration of exposure: comparing studies with exposure of less than one hour to studies with exposure longer than one hour.

We will conclude that there is a difference in effect between subgroups if the pooled effect of the group of studies with element A is significantly different from the effect of the group of studies with element B.

### 3.7 Assessing certainty of the evidence

We will use an elaborated GRADE approach (Morgan 2016) to assess the confidence in the evidence for exposure-outcome combinations. The initial level of quality of evidence is determined by the study design (‘high’ for RCT). Quality will be lowered depending on the following criteria: ROB of the underlying study, inconsistency, indirectness, imprecision and publication bias.

The following five factors are used for downgrading the quality of the body of evidence for each outcome reported in the review:

1. ROB in the evidence base can lead to downgrading with one or two levels according to the seriousness of bias. Judgement will be based on the number of studies, their impact on the meta-analysis and the seriousness of the ROB in these studies. According to the OHAT handbook, downgrading for ROB should reflect the entire body of studies included assessing a specific exposure and outcome; therefore, the decision to downgrade should be applied conservatively.
2. Inconsistency between studies means that there is a considerable difference in effect size (or direction) between studies. For example, if there are studies in the body of evidence that show a preventive effect and other studies that show a harmful effect, this indicates inconsistency or serious heterogeneity. Heterogeneity can be measured statistically using the I^2^ statistic, which varies between 0 and 100%, with 0% indicating little heterogeneity and 100% substantial heterogeneity. Because the I^2^ statistic is a relative measure, it is difficult to make a judgement of the absolute amount of heterogeneity. Therefore, it is strongly advocated to use the prediction interval (PI) estimated from the underlying distribution of effect estimates (IntHout et al., 2016). PI provides an estimate of the distribution of the true effect sizes. For an 80% PI, the true effect size for 80% of all studies falls in this interval. The reason to take an 80% PI and not a 95% PI is that the 80% PI provides a more conservative approach in making a judgement. This tells us if the exposure effect is consistent or if it varies substantially. It also tells us if the effect is harmful in all populations, or helpful in some and harmful in others. To make a judgement about the amount of heterogeneity that would be a reason for concern and a reason to downgrade if it cannot be explained, the following approach will be followed. If the 80% PI overlaps with the null value (RR = 1), it means that studies show both beneficial and harmful effects of exposure. If the 80% PI for a specific meta-analysis of RRs is of the same size as the 95% confidence interval of the mean (pooled) effect estimate, it indicates that there is no more variation in effect sizes than the statistical uncertainty. Then there is no reason for concern about heterogeneity. However, if the PI is considerably wider than the confidence interval (for example double the size) and overlaps with 1, then there is reason for concern about heterogeneity. The effect sizes of the studies vary so much that with different samples of studies the conclusions of the meta-analysis could be substantially different apart from statistical uncertainty. In this case, we will downgrade the certainty of the body of evidence by one level.
3. Indirectness refers to the extent to which PECO in the studies of the systematic review reflects the original PECO that was formulated at the start of the systematic review process. If there are considerable differences between the characteristics of those exposed to electromagnetic fields in the real world and the characteristics of those evaluated in the studies, we will downgrade the quality of the evidence by one level.
4. Imprecision. OHAT uses 95% confidence intervals as the primary method to assess imprecision. It also suggests calculating optimal information size. However, this is considered to be less informative in the scope of this review dealing with studies with complex exposure distributions in the study sample. Assumptions about the exposure distribution would have strong effects on the calculated sample size. Thus, the confidence interval is a more integrative measure of precision. We will downgrade the evidence if the upper limit of the confidence interval of a relative risk is > 2 in a non-significant effect estimate. For a significant effect estimate, downgrading is done if the upper limit of the confidence estimate divided by the point estimate is > 1.5. The OHAT handbook mentions the difficulties to distinguish between wide confidence intervals due to inconsistency and those due to imprecision. As suggested by OHAT we will prevent from downgrading twice unless if studies are both very inconsistent and imprecise.
5. Reporting bias or publication bias occurs when the publication of studies depends on the nature and direction of the results, so that the results in published studies may be systematically different from those in unpublished studies. Publication bias can thus lead to under- or overestimation of the effect of RF-EMF due to selective publication of studies. According to the OHAT handbook, some degree of publication bias is likely on any topic; however, downgrading is reserved for cases where the concern is serious enough to significantly reduce confidence in the body of evidence.

We will visually inspect the funnel plot in order to ascertain publication bias. Nevertheless, where enough studies are available per outcome (n ≥ 10), we will test publication bias based on standard meta-analytic tests (e.g. Egger's test). We will downgrade the quality in case we suspect publication bias based on such tests for publication bias. Where fewer than 10 study estimates contributed to the evidence base, we will compare study findings by publication year as there might be differences in study design parameters over time (e.g. sample sizes). We will also evaluate findings by funding source for potential risk of publication bias as previously reported for small studies sponsored by industries, non-government organizations (NGOs), or authors with conflicts of interest (Guyatt et al., 2011). Thus, we will report if there are systematic differences in the study outcome according to funding source assuming that studies with public funding are least biased compared to studies with industry funding, NGO funding or no funding source reported (Huss et al., 2007). A further indication could be identification of abstracts or other types of grey literature that do not appear as full-length articles. In case we find substantial evidence for publication bias (either by consensus among reviewers or by statistical testing), we will downgrade the confidence in the evidence quality by one unit.

No upgrading will be considered, since this review only includes randomised and cross-over trials.

The OHAT handbook suggests evaluating consistency across animal studies, across dissimilar populations and across study types. In the scope of this review, we will not consider these aspects but consistency will be evaluated at a later stage based on the outcome of the whole set of systematic reviews commissioned by the WHO.

The strength of evidence will be assessed for each outcome. The overall confidence in the association between each outcome and type of exposure is rated from high to very low.

## 4 Reporting

We will use the PRISMA guidelines for the reporting of systematic reviews to report the findings of our review (Liberati et al., 2009).

We will also include Summary of Findings table by PECO and reflecting each primary outcome.

**Financial support**

This project is funded by the World Health Organization.

WHO officials have commented on the content of the protocol, particularly on the complementarity of the different reviews commissioned.

**Author contributions**

MR has led the review team; GO drafted the first version of the protocol. MR and XBC have drafted and finalized the manuscript. SD and HJ have piloted the review instruments. JE has developed and implemented the literature search strategy. All authors have commented the various draft versions of the manuscripts.

## Funding

This work was funded by the World Health Organization (WHO).

## Declaration of Competing Interest

The authors declare the following financial interests/personal relationships which may be considered as potential competing interests: Martin Röösli’s research is entirely funded by public or not-for-profit foundations. He has served as advisor to a number of national and international public advisory and research steering groups concerning the potential health effects of exposure to nonionizing radiation, including the World Health Organization, the International Agency for Research on Cancer, the International Commission on Non-Ionizing Radiation Protection (ICNIRP), the Swiss Government (member of the working group “mobile phone and radiation” and chair of the expert group BERENIS), the German Radiation Protection Commission (member of the committee Non-ionizing Radiation (A6) and member of the working group 5G (A630)) and the Independent Expert Group of the Swedish Radiation Safety Authority. From 2011 to 2018, M.R. was an unpaid member of the foundation board of the Swiss Research Foundation for Electricity and Mobile Communication, a non-profit research foundation at ETH Zurich. Neither industry nor nongovernmental organizations are represented on the scientific board of the foundation.

Gunnhild Oftedal was appointed by a governmental body to serve on a national expert group concerning RF exposure and health hazards, is member of the core group of World Health Organization’s project assessing health effects of RF exposure and is a member of ICNIRP.

Other authors declare no other conflicts of interest.
