# Supplementary material for: The effects of radiofrequency electromagnetic fields exposure on human self-reported symptoms: A protocol for a systematic review of human experimental studies
Source: Environ Int. 2022 Jan;158:106953. doi: 10.1016/j.envint.2021.106953 (PMC8668868; doi:10.1016/j.envint.2021.106953)
Supplement: Supplementary data 1 [file mmc1.docx]

**APPENDIX 1 – Generic Search Strategy**

Ovid MEDLINE(R) and Epub Ahead of Print, In-Process & Other Non-Indexed Citations, Daily and Versions(R) <1946 to February 03, 2021> Searched 4th February 2021

This search streategy will be adapted to each specific literature database.

1 Epidemiologic studies/ (8561)

2 exp case control studies/ (1142070)

3 exp cohort studies/ (2089253)

4 ((cohort or case-control) adj (study or studies or analy*)).tw. (340827)

5 Follow-up Studies/ (656174)

6 (Follow up adj (study or studies)).tw. (50643)

7 observational study/ (92854)

8 ((observational or field) adj (study or studies)).tw. (133338)

9 Longitudinal studies/ (141898)

10 Longitudinal.tw. (260874)

11 Retrospective studies/ or Prospective studies/ (1409797)

12 (Retrospective or prospective or sham).tw. (1218146)

13 Interrupted Time Series Analysis/ (1126)

14 (time series or time point?).ti,ab. (155203)

15 ((Ecologic* or Panel) adj (study or studies)).tw. (8666)

16 "case reference".tw. (57)

17 risk factors/ or (factors adj2 risk*).tw. (1077420)

18 or/1-17 (3817334)

19 randomized controlled trial/ or equivalence trial/ or pragmatic clinical trial/ (523828)

20 controlled clinical trial/ or double-blind method/ or cross-over studies/ (276303)

21 (randomi?ed or randomly or trial or groups).ab. (2946745)

22 drug therapy.fs. (2278193)

23 ((before adj5 after) or (pre adj5 post) or pretest or pre test or posttest or post test or quasiexperiment* or quasi experiment* or double-blind* or sham or ((cross-over or crossover) adj2 (study or design or trial))).ti,ab. (764581)

24 or/19-23 (5290716)

25 telephone/ or exp cell phone/ (23179)

26 "Cell Phone Use"/ae, sn, td [Adverse Effects, Statistics & Numerical Data, Trends] (127)

27 computers, handheld/ or smartphone/ (8875)

28 ((cell* or mobile* or cordless) adj1 (phone* or telephon* or technolog* or device*)).ti,ab,kw. (20901)

29 (smartphone* or smart-phone* or cellphone* or mobiles).ti,ab,kw. (15344)

30 (ipad* or i-pad* or ipod* or i-pod* or iphone* or i-phone*).ti,ab,kw. (3043)

31 (tablet* adj3 (device* or computer*)).ti,ab,kw. (1808)

32 Magnetic Fields/ae or Electromagnetic Phenomena/ae, sn or Electromagnetic Fields/ae or Electromagnetic Radiation/ or Radio Waves/ae or Microwaves/ae, sn or Wireless Technology/lj, st, sn, td (7505)

33 (electromagnetic or electro-magnetic or radiofrequency or radio-frequency or DECT or "cordless telecomm*" or ((radio or telephon* or mobile or base or radar or broadcast* or television or tv) adj2 (station* or mast or masts or transmitter* or antenna*)) or TETRA or "plastic sealer*" or wifi or wi-fi or wlan or "wireless area network*" or "wireless network*" or radiowave* or radio-wave* or microwave* or micro-wave* or powerline* or gsm or umts or lte or 5g or mhz or ghz or "millimeter wave*" or "millimetre wave*" or "mm wave*").ti,ab,kw. (175892)

34 or/25-33 (230491)

35 Cognition/re [Radiation Effects] (473)

36 cognition disorders/ep, et, pp or auditory perceptual disorders/ep, et, pp (39376)

37 Sleep/re [Radiation Effects] (195)

38 Sleep Deprivation/ep, et, pp or Sleep Wake Disorders/ep, et, pp or Sensory Deprivation/et, ph, pp (19087)

39 memory disorders/ep, et, pp or perceptual disorders/ep, et, pp or sensation disorders/ep, et, pp or hearing disorders/ep, et, pp or somatosensory disorders/ep, et, pp or dizziness/ep, et, pp (30342)

40 Auditory Perception/ or Perception/ (64347)

41 Self Report/ (35006)

42 Fatigue/ep, et, pp or Mental Fatigue/ep, et, pp (16102)

43 Tinnitus/ep, et, pp (4659)

44 headache/ep, et, pp or psychophysiologic disorders/ep, et, pp (20486)

45 nocebo effect/ (304)

46 Hypersensitivity/ep, et, pp (11662)

47 exp Hearing Loss/ep, et, pp (29604)

48 Affective Symptoms/ep, et, pp or neurasthenia/ep, et, pp (4736)

49 (electrohypersensitiv* or IEI-EMF or "environmental intolerance" or electrosensitiv* or "electric* sensitivity" or sleep* or memory or hearing or auditory or percept* or perceiv* or self-report* or "self report*" or dizziness or dizzy or fatigue or tiredness or tinnitus or headache* or nocebo or psychophysiologic* or psychosomatic or psycho-somatic or hypersensitiv* or well-being or "well being" or "quality of life" or HRqol or (concentrat* adj2 (abilit* or inability or capacit*)) or nervous or neurasthen* or vegetative or ((body or bodily or physical or physiological* or unspecifi* or non-specifi* or indetermin* or undetermin*) adj3 (sensation* or feeling* or symptom* or change*))).ti,kw. (703090)

50 or/35-49 (854869)

51 exp animals/ not humans.sh. (4786715)

52 18 and 34 and 50 (1525)

53 52 not 51 (1463)

54 24 and 34 and 50 (2139)

55 54 not 51 (2018)

56 53 or 55 (2794)

57 (((messag* or text or texts or texting or sms or app) adj5 (treat* or therap* or cessation)) or mhealth or m-health or "mobile health" or ablation* or transcranial* or trans-cranial* or (adher* adj3 (therap* or treatment))).ti,ab,kw. (168602)

58 Text Messaging/ (3221)

59 exp Telemedicine/ (32585)

60 exp Catheter Ablation/ or exp Ablation Techniques/ or exp Radiofrequency Ablation/ or exp High-Intensity Focused Ultrasound Ablation/ (117756)

61 magnetic field therapy/ or transcranial magnetic stimulation/ (13071)

62 Transcranial Direct Current Stimulation/ or Ultrasonography, Doppler, Transcranial/ (10508)

63 or/57-62 (284497)

64 56 not 63 (2052)
