# Supplementary material for: The effects of radiofrequency electromagnetic fields exposure on human self-reported symptoms: A protocol for a systematic review of human experimental studies
Source: Environ Int. 2022 Jan;158:106953. doi: 10.1016/j.envint.2021.106953 (PMC8668868; doi:10.1016/j.envint.2021.106953)
Supplement: Supplementary data 3 [file mmc3.docx]

# Appendix xy: Risk of Bias Rating Tool for Systematic review of exposure to radiofrequency electromagnetic fields and symptoms: Evidence from human experimental studies

For the Risk of Bias assessment, we have adapted the OHAT Risk of Bias Rating Tool for Human and Animal Studies (Ref) to the specific circumstances of human experimental studies on electromagnetic field exposure and symptom development. In this document we have copied the original OHAT Risk of bias tool. In red, highlights belonging to the original OHAT tool. Any additions that we made in the instruction section to the original OHAT tool are highlighted in blue. We only kept the questions related to human controlled trials (HCT) as suggested by OHAT.

## General Instruction Format

| **How this tool is structured:** | **Study Type Abbreviations:** |
| --- | --- |
| - 11 Risk-of-bias questions or domains - Each question is applicable to 1 to 6 study design types - Questions are rated by selecting among 4 possible answers (see below) - Questions are grouped under 6 types of bias (selection, confounding, performance, attrition/exclusion, detection, and selective reporting) - In practice, we will use web-based forms and reviewers will only see questions and instructions that are relevant to the study under review (i.e., text related to human studies will   not appear during the evaluation of an animal study) | **EA:** Experimental Animal **HCT:** Human Controlled Trial^[[1]](#footnote-1)^ **Co:** Cohort  **CaCo:** Case-Control  **CrSe:** Cross-sectional  **CaS:** Case Series/Case report |

### Format of the Rating instructions:

- - Each question of the background section contains the following information
    - Definition of the general category of bias
    - Clarifying text to explain what study aspects are relevant
    - Available empirical information about the direction and magnitude of the bias
    - Information about other internal validity assessment tools that consider this element
  - Specific risk-of-bias rating instructions customized to each study type
    - Detailed criteria are outlined that define aspects of the study design, conduct, and reporting required to reach each risk-of-bias rating
    - The criteria are focused on distinguishing among the 4 risk-of-bias answers or ratings (e.g., outlining factors that separate “definitely low” from “probably low” risk of bias
  - Further explanations and instructions targeted to our review added by the study team
    - Specific criteria relevant for the available literature

### Answer Format:

***Definitely Low*** *risk of bias*:


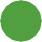


**++**

There is **direct evidence** of low risk-of-bias practices

(May include specific examples of relevant low risk-of-bias practices)

***Probably Low*** *risk of bias*:


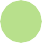


**+**

There is **indirect evidence** of low risk-of-bias practices **OR** it is deemed that **deviations from low risk-of-bias practices for these criteria during the study would not appreciably bias results, including consideration of direction and magnitude of bias**.

***Probably High*** *risk of bias*:


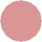

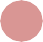


**− NR**

There is **indirect evidence** of high risk-of-bias practices **OR** there is **insufficient information (e.g., not reported or “NR”)** provided about relevant risk-of-bias practices

***Definitely High*** *risk of bias*:


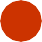


**−−**

There is **direct evidence** of high risk-of-bias practices

(May include specific examples of relevant high risk-of-bias practices)

The system for answering each risk-of-bias question requires reviewers to choose between low and high risk-of-bias options. This 4-point scale is based on the approach taken by the Clarity Group at McMaster University without an answer for mixed or unclear evidence ([2013](#_bookmark6)). A conservative approach is taken wherein insufficient information to clearly judge the risk of bias for an individual question results in an answer rating of “Probably High” risk of bias. To clearly identify answers that were reached due to insufficient information, there are two separate symbols for “Probably High” risk of bias: 1) “-“ for indirect evidence of high risk-of-bias practices, and 2) “NR” or not reported when there is insufficient information. The general answer format was adapted from ([Koustas *et al.* 2013](#_bookmark24)).

## Direction of Bias

Empirical evidence about the direction of bias is discussed for each of the risk-of-bias questions. Users of this document are encouraged to judge the direction of bias when possible. For some questions, the evidence will be easier to evaluate as toward or away from the null. For example, non-differential unintended co-exposure to high background phytoestrogen content in the diet will bias experimental studies of low-dose estrogenic effects toward the null. However, if there is no clear rationale for judging the likely direction of bias, review authors should simply outline the evidence and not attempt to guess the direction of evidence [(Sterne *et al.* 2014](#_bookmark44)).

For each bias item, we will estimate the direction of bias for the study outcome

- in favour of an effect of exposure (+)
- in favour of a null result (−)
- uncertain direction on the study outcome, i.e., in favour of either an effect of exposure or a null result (±).

### Overview of selected risk of bias questions

## Selection Bias

## Was administered dose or exposure level adequately randomized? (E)

## Was allocation to study groups adequately concealed? (E)

## Performance Bias (E)

## Were the research personnel and human subjects blinded to the study group during the study?

## Attrition/Exclusion Bias (ALL)

## Were outcome data complete without attrition or exclusion from analysis?

## Detection Bias (ALL)

## Can we be confident in the exposure characterization?

## Can we be confident in the outcome assessment?

## Selective Reporting Bias (ALL)

## Were all measured outcomes reported?

## Other Bias

1. Were there no other potential threats to **internal validity** (e.g., statistical methods were appropriate and researchers adhered to the study protocol)?
2. Funding sources (not part of OHAT)

# RISK OF BIAS RATING INSTRUCTIONS

*General remarks: Many studies in this field of research followed a crossover approach and not a parallel group approach. In a crossover study a study participant is exposed at least twice to various exposure conditions including a sham condition. These rating instructions refer mostly to a parallel group design where at least two different study groups receive different treatments or exposure. For that reason the term “study group” should be interpreted in a broad sense, and depending on the experimental study design, should be interpreted as “exposure group” or “exposure condition”.*

## Selection Bias

Selection bias refers to systematic differences between baseline characteristics of the groups that are compared [(Higgins and Green 2011](#_bookmark17)).

## Was administered dose or exposure level adequately randomized?

Randomization of exposure or sequence generation (along with allocation concealment in question #2) helps to assure that treatment is not given selectively based on potential differences in human subjects or non-human experimental animals (e.g., randomization by animal body weight avoids potential selection bias introduced by assigning all of the smallest animals to the high-dose exposure group). Randomization requires that each human subject or animal had an equal chance of being assigned to any study group including controls (e.g., use of random number table or computer generated randomization). This applies to a concurrent negative control group (i.e., a group for which exposure is to vehicle or media alone or un-treated) which must be included in the study to address randomization as well as any positive control group that may be part of the study. For some experimental designs, the analyses are performed relative to basal levels and therefore a human subject or animal may serve as its own control.

A lack of randomization can bias results away from the null towards larger effect sizes. This effect has been empirically assessed in both controlled human trials ([reviewed in Higgins and Green 2011](#_bookmark17)) and experimental animals ([reviewed in Krauth *et al.* 2013](#_bookmark26)).

This element is widely recommended to assess risk of bias for controlled human trials ([Guyatt *et al.*](#_bookmark14) [2011,](#_bookmark14) [Higgins and Green 2011,](#_bookmark17) [IOM 2011,](#_bookmark21) [Viswanathan *et al.* 2012](#_bookmark48)) and is included in most risk-of-bias instruments for animal studies (reviewed in [Krauth *et al.* 2013,](#_bookmark26) [Hooijmans *et al.* 2014](#_bookmark19)).

We recognize that given reporting practices for experimental animal studies it is unlikely that the allocation method will be explicitly reported in most studies. Thus, in cases where randomization is reported but the method is unknown (i.e., not reported and cannot be obtained through author query), we will classify studies as “probably low risk of bias”. In cases where randomization is not reported, we will assume that randomization was not undertaken and classify such studies as “probably high risk of bias”.

*Note: normalization is discussed in a separate risk-of-bias question under confounding bias: Did the study design or analysis account for important confounding or modifying variables?*

*For a crossover study adequate randomization means that allocation of sessions is counterbalanced, as otherwise learning effects may bias the outcome of the study.*

## Applies to: HCT, EA Definitely Low risk of bias:

**HCT**: There is direct evidence that subjects were allocated to any study group including controls using a method with a random component. Acceptable methods of randomization include: referring to a random number table, using a computer random number generator, coin tossing, shuffling cards or envelopes, throwing dice, or drawing of lots [(Higgins and Green 2011](#_bookmark17)). Restricted randomization (e.g., blocked randomization) to ensure particular allocation ratios will be considered low risk of bias. Similarly, stratified randomization and minimization approaches that attempt to minimize imbalance between groups on important prognostic factors (e.g., body weight) will be considered acceptable. There is direct evidence for crossover study that allocation of sessions was counterbalanced

## Probably Low risk of bias:

**HCT**: There is indirect evidence that subjects were allocated to study groups using a method with a random component (i.e., authors state that allocation was random, without description of the method used),

**OR** it is deemed that allocation without a clearly random component during the study would not appreciably bias results. For example, approaches such as biased coin or urn randomization, replacement randomization, mixed randomization, and maximal randomization may require consultation with a statistician to determine risk-of-bias rating [(Higgins and Green 2011](#_bookmark17)). In a crossover study, no appreciably bias from imperfect counterbalancing is expected to occur, if the sequence number is included in the data analysis.

## Probably High risk of bias:

**HCT**: There is indirect evidence that subjects were allocated to study groups using a method with a non-random component,
**OR** in a crossover study sequence of sessions was not counterbalanced and this was not considered in the data analysis.

**OR** there is insufficient information provided about how subjects were allocated to study groups (record “NR” as basis for answer).

**Note:** Non-random allocation methods may be systematic, but have the potential to allow participants or researchers to anticipate the allocation to study groups. Such “quasi-random” methods include alternation, assignment based on date of birth, case record number, or date of presentation to study [(Higgins and Green 2011](#_bookmark17)).

## Definitely High risk of bias:

**HCT**: There is direct evidence that subjects were allocated to study groups using a non-random method including judgment of the clinician, preference of the participant, the results of a laboratory test or a series of tests, or availability of the intervention ([Higgins and Green 2011](#_bookmark17)). In a crossover study definitive high risk of bias occurs when always the same order of exposure conditions was applied.

## Was allocation to study groups adequately concealed?

Allocation concealment prior to assigning the exposure level or treatment group (along with randomization in question #1) helps to assure that treatment is not given selectively based on potential differences in human subjects or non-human experimental animals.

Allocation concealment requires that research personnel allocating subjects or animals to treatment groups (including the control group) could not foresee which administered dose or exposure level is going to be assigned at the start of a study. Human studies also require that allocation be concealed from human subjects prior to entering the study.

A lack of allocation concealment can bias results away from the null towards larger effect sizes. This effect has been empirically assessed in both controlled human trials [[(Schulz *et al.* 1995,](#_bookmark39) [Schulz *et al.*](#_bookmark40) [2002,](#_bookmark40) [Pildal *et al.* 2007](#_bookmark36)); see also studies reviewed in ([Higgins and Green 2011](#_bookmark17))] and in animal studies [([Macleod *et al.* 2008](#_bookmark28)) ; see also studies reviewed in ([Krauth *et al.* 2013](#_bookmark26))].

This element is widely recommended to assess risk of bias for controlled human trials ([Guyatt *et al.*](#_bookmark14) [2011,](#_bookmark14) [Higgins and Green 2011,](#_bookmark17) [IOM 2011,](#_bookmark21) [Viswanathan *et al.* 2012](#_bookmark48)) and included in some risk-of-bias instruments for animal studies ([reviewed in Krauth *et al.* 2013](#_bookmark26)).

*Note: there are separate risk-of-bias issues affected by concealment or blinding that are important for selection, performance and detection bias: 1) a question under performance bias addresses blinding of research personnel and human subjects to study groups during the study (addressed in question 4); and 2) a question under detection bias addresses blinding during outcome assessment (addressed below in question67).*

Note that a crossover study cannot suffer from concealing bias, because study participants will be exposed to all exposure conditions and thus there is no a priori knowledge to be shared at the time of recruitment.

## Definitely Low risk of bias:

**HCT**: There is direct evidence that at the time of recruitment the research personnel and subjects did not know what study group subjects were allocated to, and it is unlikely that they could have broken the blinding of allocation until after recruitment was complete and irrevocable. Acceptable methods used to ensure allocation concealment include central allocation (including telephone, web-based and pharmacy-controlled randomization); sequentially numbered drug containers of identical appearance; sequentially numbered, opaque, sealed envelopes; or equivalent methods. $

## Probably Low risk of bias:

**HCT**: There is indirect evidence that the research personnel and subjects did not know what study group subjects were allocated to and it is unlikely that they could have broken the blinding of allocation until after recruitment was complete and irrevocable,

**OR** it is deemed that lack of adequate allocation concealment would not appreciably bias results.

## Probably High risk of bias:

**HCT**: There is indirect evidence that at the time of recruitment it was possible for the research personnel and subjects to know what study group subjects were allocated to, or it is likely that they could have broken the blinding of allocation before recruitment was complete and irrevocable,

**OR** there is insufficient information provided about allocation to study groups (record “NR” as basis for answer).

**Note:** Inadequate methods include using an open random allocation schedule (e.g., a list of random numbers); assignment envelopes used without appropriate safeguards (e.g., if envelopes were unsealed or non-opaque or not sequentially numbered); alternation or rotation; date of birth; case record number; or any other explicitly unconcealed procedure. For example, if the use of assignment envelopes is described, but it remains unclear whether envelopes were sequentially numbered, opaque and sealed.

## Definitely High risk of bias:

**HCT**: There is direct evidence that at the time of recruitment it was possible for the research personnel and subjects to know what study group subjects were allocated to, or it is likely that they could have broken the blinding of allocation before recruitment was complete and irrevocable.

## Were the research personnel and human subjects blinded to the study group during the study?

Blinding requires that research personnel do not know which administered dose or exposure level the human subject or animal is being given (i.e., study group). Human studies also require blinding of the human subjects when possible.

**Human introductory text:** If research personnel or human subjects are not blinded to the study groups it could affect the actual outcomes of the participants due to differential behaviors across intervention groups. During the course of a study blinding of participants and research personnel is a recommended risk-of-bias element in the most recent Cochrane guidance for assessing randomized clinical trials [(Higgins and Green 2011](#_bookmark17)).

No empirical evidence of bias due to failure to blind during the course of a study is currently available. However, ‘blind’ or ‘double-blind’ study descriptions usually include blinding of research personnel, human subjects, or both. Without distinguishing between the different stages of blinding during the conduct of a study, lack of blinding in randomized trials has been empirically shown to be associated with larger estimations of intervention effects (on average a 9% increase in an odds ratio) ([Pildal *et al.*](#_bookmark36) [2007](#_bookmark36)). Schulz *et al*. ([1995](#_bookmark39)) analyzed 250 controlled trials and found that studies that were not double- blinded had a 17% larger estimation of treatment effect, on average. If additional investigations or co- interventions occur differentially across intervention groups, bias can also be introduced by not blinding research personnel or human subjects.

For some exposures, it is not possible to entirely blind research personnel and subjects during the course of the study (an exercise intervention or patients receiving surgery). However, adherence to a strict study protocol to minimize differential behaviors by research personnel and human subjects can reduce the risk of bias. In practice, successful blinding cannot be ensured, as it can be compromised for most interventions. In some case the treatment may have side effects possibly allowing the participant to detect which intervention they received, unless the study compares interventions with similar side effects or uses an active placebo [(Boutron *et al.* 2006](#_bookmark4)).

It is important to realize that for this specific topic on self-reported outcome, adequate blinding is a very crucial aspect for the risk of bias analysis. In a crossover study, participants may register minimal differences between various exposure conditions which could be indicative for the exposure status such as sound or vibration generated by the exposure set-up. Ideally, a study has conducted tests, whether exposure conditions can be perceived or not.

## Definitely Low risk of bias:

**HCT**: There is direct evidence that the subjects and research personnel were adequately blinded to study group, and it is unlikely that they could have broken the blinding during the study. Methods used to ensure blinding include central allocation; sequentially numbered drug containers of identical appearance; sequentially numbered, opaque, sealed envelopes; or equivalent methods. Such a study is labelled double-blind. It has been reported that the exposure set-up was tested in terms of perceivable emissions.

## Probably Low risk of bias:

**HCT**: There is indirect evidence that the research personnel and subjects were adequately blinded to study group, and it is unlikely that they could have broken the blinding during the study,

**OR** it is deemed that lack of adequate blinding during the study would not appreciably bias results. In case no information about perception of RF-EMF exposure is given and it is deemed to be unlikely that exposure conditions can be perceived. An example for low risk of bias would be a single-blinded study, where the researcher but not the study participant is aware of the exposure status but the type of interaction with study participants is deemed unlikely to result in a bias.

## Probably High risk of bias:

**HCT**: There is indirect evidence that it was possible for research personnel or subjects to infer the study group. This could be the case if for a given exposure set-up there is evidence that it generates some kind of noise or vibration that can be perceived.

**OR** there is insufficient information provided about blinding to study group during the study (record “NR” as basis for answer).

**Note:** Inadequate methods include using an open random allocation schedule (e.g., a list of random numbers), assignment envelopes used without appropriate safeguards (e.g., if envelopes were unsealed or non-opaque or not sequentially numbered), alternation or rotation; date of birth; case record number; or any other explicitly unconcealed procedure. For example, if the use of assignment envelopes is described, but it remains unclear whether envelopes were sequentially numbered, opaque and sealed.

## Definitely High risk of bias:

**HCT**: There is direct evidence for lack of adequate blinding of the study group including no blinding or incomplete blinding of research personnel and subjects. For some treatments, such as behavioral interventions, allocation to study groups cannot be concealed.

## Attrition/Exclusion Bias (ALL)

Attrition or exclusion bias refers to systematic differences in the loss or exclusion from analyses of participants or animals from the study and how they were accounted for in the results [(Viswanathan *et*](#_bookmark48) [*al.* 2012](#_bookmark48)).

## Were outcome data complete without attrition or exclusion from analysis?

Incomplete outcome data includes loss due to attrition (nonresponse, dropout, or loss to follow-up) or exclusion from analyses. The degree of bias resulting from incomplete outcome data depends on the reasons that outcomes are missing, the amount and distribution of missing data across groups, and the potential association between outcome values and likelihood of missing data ([Higgins and Green 2011](#_bookmark17)). The risk of bias from incomplete outcome data can be reduced if study authors address the problem in their analyses (e.g., intention to treat analysis and imputation). Exclusion of individuals or animals from analyses should be clearly reported and outliers identified with appropriate statistical procedures.

**Human introductory text:** Differential or overall attrition because of nonresponse, dropping out, loss to follow-up, and exclusion of participants can introduce bias when missing outcome data are related to both exposure/treatment and outcome. Those who drop out of the study or who are lost to follow-up may be systematically different from those who remain in the study. Attrition or exclusion bias can potentially change the collective (group) characteristics of the relevant groups and their observed outcomes in ways that affect study results by confounding and spurious associations [(Viswanathan *et al.*](#_bookmark48) [2012](#_bookmark48)). This risk-of-bias element is recommended to assess controlled human trials ([Higgins and Green](#_bookmark17) [2011](#_bookmark17)), observational human studies ([Viswanathan *et al.* 2012,](#_bookmark48) [Sterne *et al.* 2014](#_bookmark44)) and animal studies ([Krauth *et al.* 2013](#_bookmark26)). However, concern over bias from incomplete outcome data is mainly theoretical and most studies that have looked at whether aspects of missing data are associated with magnitude of effect estimates have not found clear evidence of bias ([reviewed in Higgins and Green 2011](#_bookmark17)).

In this field of research a concern is that individuals reacting strongly RF-EMF exposure (e.g. EHS individuals) are discontinuing the experiment due to unpleasant experiences. In particular crossover studies that require patients to present them several times, may be vulnerable to this kind of bias.

## Definitely Low risk of bias:

**HCT**: There is direct evidence that there was no loss of subjects during the study and outcome data were complete,

**OR** loss of subjects (i.e., incomplete outcome data) was adequately addressed and reasons were documented when human subjects were removed from a study or analyses. Review authors should be confident that the participants included in the analysis are exactly those who were randomized into the trial. Acceptable handling of subject attrition includes: very little missing outcome data (less than 10% in each group [(Genaidy *et al.* 2007](#_bookmark11))); reasons for missing subjects unlikely to be related to outcome (for survival data, censoring unlikely to be introducing bias); missing outcome data balanced in numbers across study groups, with similar reasons for missing data across groups,

**OR** analyses (such as intention-to-treat analysis) in which missing data have been imputed using appropriate methods (insuring that the characteristics of subjects lost to follow up or with unavailable records are described in identical way and are not significantly different from those of the study participants).

**Note:** Participants randomized but subsequently found not to be eligible need not always be considered as having missing outcome data [(Higgins and Green 2011](#_bookmark17)).

## Probably Low risk of bias:

**HCT**: There is indirect evidence that loss of subjects (i.e., incomplete outcome data) was adequately addressed and reasons were documented when human subjects were removed from a study,

**OR** it is deemed that the proportion lost to follow-up would not appreciably bias results (less than 20% in each group ([Genaidy *et al.* 2007](#_bookmark11))). This would include reports of no statistical differences in characteristics of subjects lost to follow up or with unavailable records from those of the study participants. Generally, the higher the ratio of participants with missing data to participants with events, the greater potential there is for bias. For studies with a long duration of follow-up, some withdrawals for such reasons are inevitable.

## Probably High risk of bias:

**HCT**: There is indirect evidence that loss of subjects (i.e., incomplete outcome data) was unacceptably large (greater than 20% in each group [(Genaidy *et al.* 2007](#_bookmark11))) and not adequately addressed,

**OR** there is insufficient information provided about numbers of subjects lost to follow-up (record “NR” as basis for answer).

## Definitely High risk of bias:

**HCT**: There is direct evidence that loss of subjects (i.e., incomplete outcome data) was unacceptably large and not adequately addressed. Unacceptable handling of subject attrition includes: reason for missing outcome data likely to be related to true outcome, with either imbalance in numbers or reasons for missing data across study groups; or potentially inappropriate application of imputation.

## Detection Bias (ALL)

Detection bias refers to systematic differences between experimental and control groups with regards to how outcomes and exposures are assessed ([Higgins and Green 2011](#_bookmark17)) and also considers validity and reliability of methods used to assess outcomes and exposures ([Viswanathan *et al.* 2012](#_bookmark48)).

## Can we be confident in the exposure characterization?

Confidence in the exposure requires valid, reliable, and sensitive methods to measure exposure applied consistently across groups. Exposure misclassification or measurement error may be independent of the outcomes (non-differential) or related to the outcome of interest (differential). Non-differential measurement error of exposures will usually bias the results toward the null by lowering precision and therefore reducing the ability to distinguish potential effects between exposure levels. Therefore, this tool considers the accuracy of the exposure characterization, including both purity and stability for controlled exposure studies, as part of the risk-of-bias rating for exposure. Differential measurement error of exposures can bias the exposure-outcome relationship and result in detection bias.

Detection bias can be minimized by using valid and reliable exposure measures applied consistently across groups (i.e., under the same method and time-frame). Studies that directly measure exposure in subjects (e.g., measurement of the chemical in blood, plasma, urine, etc.) are likely to have less measurement error and less risk of bias for exposure than studies relying on indirect measures (e.g., predictions from activity patterns and microenvironment concentrations). Exposure information obtained by self-report depends on the recall of participants and differential errors in recall can attenuate, strengthen, or even invert the true relationship ([White 2003](#_bookmark51)). Self-reporting of exposures for case-control studies are frequently cited as leading to differential measurement errors because cases often remember past exposures better than controls (i.e., recall bias) ([e.g., see Rothman *et al.* 2012](#_bookmark38)). Differential measurement error could also be introduced if the exposure data for different groups come from different sources for observational studies or are taken at different time points for experimental studies.

Acceptable methods for measuring exposure will be highly exposure dependent and therefore a specific list of acceptable, inaccurate, or potentially biased methods should be developed for each evaluation and will require subject-matter expertise. It is recommended that experts with some knowledge of the literature (including exposure and outcomes) participate in drafting the risk-of-bias criteria for exposure characterization when a review protocol is developed. Even with early expert consultation and planning, exposure questions may arise when the actual studies are assessed. Additional consultation and modifications to the exposure risk-of-bias criteria may be necessary. When changes are made, they should be documented along with the date on which modifications were made and the logic for the changes.

For controlled exposure studies (i.e., experimental human or animal studies), the use of reliable methods to measure exposure depends primarily on ensuring the purity and stability of the treatment compound. Independent verification of purity would be considered best practice because the identity and purity as listed on the bottle can be inaccurate. In NTP’s experience, about 3% of chemicals purchased are the wrong chemical and the inaccuracy rate of chemical labelling rises to 10% if you include inaccurate reporting of purity (unpublished, personal communication Brad Collins, NTP chemist). It is also possible that impurities may be more toxic than the compound of interest. This occurred during an NTP study of PCB 118 where analysis revealed the presence of 0.622% of the much more potent PCB 126, resulting in the study being continued as a mixture study [[(NTP 2006](#_bookmark31)), see page 13]. The directions below takes a conservative approach in requiring independent verification of ≥99% purity for a single substance for “definitely low” risk of bias. However, the risk of bias associated with exposure to impurities depends on the identity of the impurities and the sensitivity of the outcome of interest which could result in potential effects of those impurities on the outcome of interest. The threshold for these values should be developed for specific research questions and reflect empirical data for the substance and outcome under consideration when possible. Therefore, for some chemicals like PCBs, ≥99% purity may not be sufficient for “definitely low” risk of bias and for others the appropriate purity value may be lower.

Exposure characterization should also include verification of the compound over the course of the test period. This is particularly important if the compound is volatile or instable. For example, daily preparation of treatment solutions may be required for unstable compounds (e.g., half-lives on the order of days). Special apparatus such as flow-through systems are needed to ensure exposure to volatile compounds. For example, Durda and Preziosi [(2000](#_bookmark9)) suggest the use of flow-through systems in aquatic exposures to volatile compounds (e.g., those with Henry’s Law values in the range of 10^-5^ atm- m^3^/mol or greater).

**Human introductory text:** Assessment of exposure is a widely used element of tools to assess study quality for observational human studies [(Downs and Black 1998](#_bookmark7), [Shamliyan *et al.* 2010](#_bookmark42), [Viswanathan *et*](#_bookmark48) [*al.* 2012,](#_bookmark48) [CLARITY Group at McMaster University 2013](#_bookmark6), [Wells *et al.* 2014](#_bookmark50)). Exposure is much more difficult to measure and to accurately ensure for observational studies than for controlled exposure studies. Therefore, exposure measurement error and misclassification are more likely to contribute to risk of bias for observational studies.

The direction of the bias (towards or away from the null) will differ based on the nature of differences between comparison groups and may be difficult to predict. Non-differential misclassification of exposure will generally bias results towards the null, but differential misclassification can bias towards or away from the null, making it difficult to predict the direction of effect ([Szklo and Nieto 2007](#_bookmark45)). For controlled exposure studies, noncompliance with the allocated treatment could introduce differential misclassification if compliance was unequal across study groups. Adherence to a strict study protocol that includes measures to assure or assess compliance can reduce the risk of bias.

A common concern in this field of research is to use RF-EMF emitting devices, without controlling the exposure status. It has to be noted that transmission rate of such devices can vary a lot depending on various factors such as network quality or data rate. Mobile phones may even stop Rf-EMF emissions if there is no sound to transmit.

“Well-established” exposure assessment methods in the this field of research include appropriate numerical computations of SAR inside the body of anatomical body models using validated numerical source models. For the specific case where the brain is considered the target organ, localized exposure of the head with the radiation source close to the ear, only indicating maximum spatial average SAR (SAR10g, SAR1g) in the entire head (i.e., including ear tissue) is considered insufficient, as the SAR1g and/or the SAR10g value may be highly dominated by the absorption inside the ear and can therefore not be seen as a reliable proxy for brain exposure. Similarly, only indicating SAR10g or SAR1g assessed in a homogeneous head phantom is considered insufficient in case of localized exposure of the head with the radiation source close to the ear.

“Less-established” exposure assessment methods in the above sense include the assessment of exposure by the undisturbed (i.e. measured in absence of the subject) external electric or magnetic field strengths (or power flux density) at the site of the subject, which can be transferred to SAR levels in case of far field conditions. Tissue external exposure measures (electric or magnetic field strengths or power flux density) at the site of the subject is considered adequate for far-field exposure in a homogeneous field. However, for near-field exposure studies it is insufficient to only report external field quantities.

“Puritiy” in the sense below means that background RF-EMF exposure has been evaluated and is considered to be less than 1% of the lowest applied exposure conditions.

## Definitely Low risk of bias:

**HCT, EA**: There is direct evidence that the exposure (including purity and stability of the test substance and compliance with the treatment, if applicable) was independently characterized and purity confirmed generally as ≥99%^[[2]](#footnote-2)^ for single substance or non-mixture evaluations ([see](#_bookmark31) [NTP 2006 for example of study effects attributable to impurities of approximately 1%](#_bookmark31)),

**AND** that exposure was consistently administered (i.e., with the same method and time-frame) across treatment groups.

**Note:** This means that a well controlled exposure apparatus was used for all considered exposure levels, and that the resulting exposure of the subjects were assessed using well-established methods that are directly related to SAR level inside the body or the relevant target organ for the outcome to be considered,

**OR** exposure was assessed using less-established methods that are validated against SAR models that represent the investigated exposure situation.

**AND** the exposure distribution inside the body or relevant organ contains sufficient contrast between the lowest and the highest exposure categories, when taking into account intra- and inter-individual variations of exposure (in terms of SAR).

## Probably Low risk of bias:

**HCT, EA**: There is indirect evidence that the exposure (including purity and stability of the test substance and compliance with the treatment, if applicable) was independently characterized and purity confirmed generally as ≥99%^3^ (i.e., the supplier of the chemical provides documentation of the purity of the chemical),

**OR** direct evidence that purity was independently confirmed as ≥98%^3^ it is deemed that impurities of up to 2% would not appreciably bias results,

**AND** there is indirect evidence that exposure was consistently administered (i.e., with the same method and time-frame) across treatment groups.

**Note:** This means that there is indirect evidence that the exposure was consistently generated and assessed in the sense described above

**AND** the exposure distribution inside the brain contains sufficient contrast between the lowest and the highest exposure categories, when taking into account intra- and inter-individual variations of exposure (in terms of SAR).

## Probably High risk of bias:

**HCT, EA**: There is indirect evidence that the exposure (including purity and stability of the test substance and compliance with the treatment, if applicable) was assessed using poorly validated methods,

**OR** there is insufficient information provided about the validity of the exposure assessment method, but no evidence for concern (record “NR” as basis for answer).

**Note:** This means that there is indirect evidence that the exposure was generated and assessed using poorly validated methods (e.g. indicating SAR1g or SAR10g values inside homogeneous head phantom in case of localized exposure of the head with the radiation source close to the ear),

**OR** there is insufficient information provided about the exposure generation and assessment, but no evidence for concern about the method used (record “NR” as basis for answer).

**OR** the exposure contrast is probably too low to ensure a non-overlapping of lowest and highest exposure categories when taking into account intra- and inter-individual variations of exposure (in terms of SAR).

## Definitely High risk of bias:

**HCT, EA**: There is direct evidence that the exposure (including purity and stability of the test substance and compliance with the treatment, if applicable) was assessed using poorly validated methods.

**Note:** There is direct evidence that exposure generation and assessment is done with poorly validated methods (e.g. indicating SAR1g or SAR10g values inside homogeneous head phantom in case of localized exposure of the head with the radiation source close to the ear; lack of control/monitoring of exposure), ¨

**OR** there is direct evidence that the exposure contrast is too low to ensure a non-overlapping of lowest and highest exposure categories when taking into account intra- and inter-individual variations of exposure (in terms of SAR).

**OR** studies using near field exposure and reporting only tissue-external exposure metrics will also be rated definitely high risk of bias

## Can we be confident in the outcome assessment?

Confidence in the outcome requires valid, reliable, and sensitive methods to assess the outcome applied consistently across groups. Outcome misclassification or measurement error may be unrelated to the exposure (non-differential) or related to the exposure (differential). Non-differential measurement error of outcomes will usually bias the results toward the null by lowering precision and therefore reducing the ability to distinguish potential effects on exposure between exposure levels. Differential measurement error of outcomes can bias the exposure-outcome relationship and result in detection bias. There are three important factors for assessing bias in the outcome assessment: 1) the objectivity of the outcome assessment, 2) consistency in measurement of outcomes, and 3) blinding of the outcome assessors (for knowledge of the exposure).

Detection bias can be minimized by using valid and reliable methods to assess the outcome applied consistently across groups (i.e., under the same method and time-frame). Objectivity of the outcome assessment and the need for blinding are two sides of the same issue. Blinding requires that outcome assessors do not know the study group or exposure level of the human subject or animal when the outcome was assessed. The objectivity of procedures used for measuring and reporting an outcome will impact the degree to which outcome assessors could bias the reported results. For example, a behavioral outcome rated by a researcher (i.e., direct observation of behaviors) relies on subjective judgment and therefore may be impacted by potential bias of the outcome assessor to a greater degree than outcomes that are measured by machines (e.g., automated red blood cell counts). Similarly, studies relying on self-report of outcome may be rated as having a higher risk of bias than studies with clinically observed outcomes [(Viswanathan *et al.* 2012](#_bookmark48)). Although objective measures are less prone to bias by researchers than subjective measures, bias could be introduced during sample preparation or handling and therefore blinding still has a role in controlling for potential bias unless sample preparation and outcome measurement are accomplished with automated procedures. For example, the potential for outcome assessors to introduce bias would be minimized for *ex vivo* studies where samples are collected and outcomes are assessed automatically within an apparatus.

Acceptable methods for measuring the outcomes of interest will be highly dependent on the outcome and therefore a specific list of acceptable, inaccurate, or potentially biased methods should be developed for each evaluation and will require subject-matter expertise. It is recommended that experts with some knowledge of the literature (including both exposure and outcome) participate in drafting the risk-of-bias criteria for outcome assessment when a review protocol is developed. Even with early expert consultation and planning, outcome questions may arise when the actual studies are assessed because of non-traditional methods, application to non-traditional species, or endpoints that are indirectly related to the outcome of interest. Additional consultation and modifications to the outcome risk-of-bias criteria may be necessary. When changes are made, they should be documented along with the date on which modifications were made and the logic for the changes.

**Human introductory text:** Differential methods used in the assessment of outcomes is a source of bias and this is a widely used risk-of-bias element in tools for observational human studies ([Downs and Black](#_bookmark7) [1998,](#_bookmark7) [Genaidy *et al.* 2007,](#_bookmark11) [Shamliyan *et al.* 2010](#_bookmark42), [Viswanathan *et al.* 2012](#_bookmark48), [Sterne *et al.* 2014](#_bookmark44)). The recent guidance for non-randomized studies of interventions suggests considering the objectivity of the outcome assessment when evaluating bias in the outcome assessment ([Sterne *et al.* 2014](#_bookmark44)) and we have included consideration of the objectivity in this document for evaluating the potential impact of blinding practices. Blinding of outcome assessors is a widely recommended risk-of-bias element for controlled trials and observational studies ([Higgins and Green 2011,](#_bookmark17) [Viswanathan *et al.* 2012,](#_bookmark48) [Sterne *et al.* 2014](#_bookmark44)). For human studies blinding of the subject to exposure levels should also be considered. For example, a subject’s knowledge of their own exposure levels would represent an increased risk of bias for self- reported outcomes relative to clinically measured outcomes.

Without distinguishing between the different stages of blinding during the conduct of a study, lack of blinding in randomized trials has been empirically shown to be associated with larger estimations of intervention effects (on average a 9% increase in an odds ratio) ([Pildal *et al.* 2007](#_bookmark36)). Schulz et al. ([1995](#_bookmark39)) analyzed 250 controlled trials and found that studies that were not double-blinded had a 17% larger estimation of treatment effect, on average. In trials with more subjective outcomes, more bias has been observed with lack of blinding ([Wood *et al.* 2008](#_bookmark52)), indicating that blinding outcome assessors could be more important for these effects.

For some exposures, it is not possible to entirely blind outcome assessors, particularly if subjects are self-reporting outcomes. In practice, successful blinding cannot always be ensured, as it can be compromised for most interventions. In some cases the treatment may have side effects possibly allowing the participant to detect which intervention they received, unless the study compares interventions with similar side effects or uses an active placebo ([Boutron *et al.* 2006](#_bookmark4)).

The nature of these outcomes means that primarily self-reported symptoms, including any bodily sensation or a feeling or change in well-being which is obtained by a written questionnaire or personal interview. We will consider as ‘reliable’ the use of methods, scales, scores, which authors claim and referenced as having been validated and sensitive to short term effects.

## Definitely Low risk of bias:

**HCT, Co**: There is direct evidence that the outcome was assessed using well-established methods [(e.g., the “gold standard” with validity and reliability >0.70 Genaidy *et al.* 2007](#_bookmark11)),

**AND** subjects had been followed for the same length of time in all study groups. Acceptable assessment methods will depend on the outcome, but examples of such methods may include: objectively measured with diagnostic methods, measured by trained interviewers, obtained from registries [(Shamliyan *et al.* 2010](#_bookmark42)) and written questionnaires,

**AND** there is direct evidence that the outcome assessors (including study subjects, if outcomes were self-reported) were adequately blinded to the study group, and it is unlikely that they could have broken the blinding prior to reporting outcomes.

## Probably Low risk of bias:

**HCT, Co**: There is indirect evidence that the outcome was assessed using acceptable methods (i.e., deemed valid and reliable but not the gold standard) [(e.g., validity and reliability ≥0.40 Genaidy](#_bookmark11) [*et al.* 2007](#_bookmark11)),

**AND** subjects had been followed for the same length of time in all study groups [Acceptable, but not ideal assessment methods will depend on the outcome, but examples of such methods may include proxy reporting of outcomes and mining of data collected for other purposes],

**OR** it is deemed that the outcome assessment methods used would not appreciably bias results,

**AND** there is indirect evidence that the outcome assessors (including study subjects, if outcomes were self-reported) were adequately blinded to the study group, and it is unlikely that they could have broken the blinding prior to reporting outcomes,

**OR** it is deemed that lack of adequate blinding of outcome assessors would not appreciably bias results, which is more likely to apply to objective outcome measures.

## Probably High risk of bias:

**HCT, Co**: There is indirect evidence that the outcome assessment method is an insensitive instrument (e.g., a questionnaire used to assess outcomes with no information on validation),

**OR** the length of follow up differed by study group,

**OR** there is indirect evidence that it was possible for outcome assessors (including study subjects if outcomes were self-reported) to infer the study group prior to reporting outcomes,

**OR** there is insufficient information provided about blinding of outcome assessors (record “NR” as basis for answer).

## Definitely High risk of bias:

**HCT, Co**: There is direct evidence that the outcome assessment method is an insensitive instrument,

**OR** the length of follow up differed by study group,

**OR** there is direct evidence for lack of adequate blinding of outcome assessors (including study subjects if outcomes were self-reported), including no blinding or incomplete blinding.

## Selective Reporting Bias (ALL)

Selective reporting bias refers to selective inclusion of outcomes in the publication of the study on the basis of the results ([Hutton and Williamson 2000,](#_bookmark20) [Higgins and Green 2011](#_bookmark17)).

## Were all measured outcomes reported?

Selective reporting of results is a recommended element of assessing risk of bias ([Guyatt *et al.* 2011,](#_bookmark14) [Higgins *et al.* 2011,](#_bookmark18) [IOM 2011,](#_bookmark21) [Viswanathan *et al.* 2012](#_bookmark48)). Selective reporting is present if pre-specified outcomes are not reported or incompletely reported. It is likely widespread and difficult to assess with confidence for most studies unless the study protocol is available. Selective reporting bias can be assessed by comparing the “methods” and “results” section of the paper, and by considering outcomes measured in the context of knowledge in the field. Abstracts of presentations relating to the study may contain information about outcomes not subsequently mentioned in publications. Selective reporting bias should be suspected if the study does not report outcomes in the results section that would have been expected based on the methods, or if a composite score is present without the individual component outcomes ([Guyatt *et al.* 2011](#_bookmark14)). It may be useful to pay attention to author affiliations and funding source which can contribute to selective outcome reporting when results are not consistent with expectations or value to the research objectives.

## Definitely Low risk of bias:

**HCT, EA, Co, CaCo, CrSe, CaS**: There is direct evidence that all of the study’s measured outcomes (primary and secondary) outlined in the protocol, methods, abstract, and/or introduction (that are relevant for the evaluation) have been reported. This would include outcomes reported with sufficient detail to be included in meta-analysis or fully tabulated during data extraction and analyses had been planned in advance.

## Probably Low risk of bias:

**HCT, EA, Co, CaCo, CrSe, CaS**: There is indirect evidence that all of the study’s measured outcomes (primary and secondary) outlined in the protocol, methods, abstract, and/or introduction (that are relevant for the evaluation) have been reported,

**OR** analyses that had not been planned in advance (i.e., retrospective unplanned subgroup analyses) are clearly indicated as such and it is deemed that the unplanned analyses were appropriate and selective reporting would not appreciably bias results (e.g., appropriate analyses of an unexpected effect). This would include outcomes reported with insufficient detail such as only reporting that results were statistically significant (or not).

## Probably High risk of bias:

**HCT, EA, Co, CaCo, CrSe, CaS**: There is indirect evidence that all of the study’s measured outcomes (primary and secondary) outlined in the protocol, methods, abstract, and/or introduction (that are relevant for the evaluation) have been reported,

**OR** and there is indirect evidence that unplanned analyses were included that may appreciably bias results,

**OR** there is insufficient information provided about selective outcome reporting (record “NR” as basis for answer).

## Definitely High risk of bias:

**HCT, EA, Co, CaCo, CrSe, CaS**: There is direct evidence that all of the study’s measured outcomes (primary and secondary) outlined in the protocol, methods, abstract, and/or introduction (that are relevant for the evaluation) have not been reported. In addition to not reporting outcomes, this would include reporting outcomes based on composite score without individual outcome components or outcomes reported using measurements, analysis methods or subsets of the data (e.g., subscales) that were not pre-specified or reporting outcomes not pre-specified, or that unplanned analyses were included that would appreciably bias results.

## Other Bias

1. **Were there no other potential threats to internal validity (e.g., statistical methods were appropriate and researchers adhered to the study protocol)?**

On a project specific basis, additional questions for other potential threats to internal validity can be added and applied to study designs as appropriate.

## Were statistical methods appropriate?

Some of the more extensive quality tools have a separate question for appropriateness of the statistical methods [(e.g., 1 of the 25 elements in the Downs and Black 1998 tool addresses the statistics](#_bookmark7)); however most do not include a separate question. The OHAT risk-of-bias tool suggests consideration of statistical methods with the other potential threats to internal validity. One of the common statistical issues identified has been reporting of statistical tests that require normally distributed data (e.g., t-test or ANOVA) without reporting that the homogeneity of variance was tested or confirmed.

It is recommended that experts with some knowledge of statistical methods used in the literature participate in drafting the risk-of-bias criteria for identifying inappropriate statistical methods when a review protocol is developed. Even with early expert consultation and planning, statistical methods questions may arise when the actual studies are assessed. Additional consultation and modifications to the statistical methods risk-of-bias criteria may be necessary. When changes are made, they should be documented along with the date on which modifications were made and the logic for the changes.

Symptom score data may rarely follow a normal distribution but rather a negative binomial or Poisson distribution. It should thus be checked whether adequate statistical methods have been used.

## Did researchers adhere to the study protocol?

Not applicable

## Did the study design or analysis account for important confounding and modifying variables (including unintended co-exposures) in experimental studies?

There is a separate risk-of-bias question to address confounding and modifying variables (including co- exposures) for observational studies because confounding is a much greater concern for observational studies. Controlled exposure studies (i.e., experimental human or animal studies) can address confounding through study design features such as randomization and allocation concealment.

Therefore, most study quality tools for experimental studies do not include questions for confounding ([Higgins *et al.* 2011,](#_bookmark18) [Krauth *et al.* 2013,](#_bookmark26) [Koustas *et al.* 2014](#_bookmark25)). Confounding by chance (i.e., confounding that is unknown, unmeasured, or poorly measured) is expected to be equally distributed between groups under true randomization; however, experimental studies may not always successfully randomize potential confounders ([Viswanathan *et al.* 2013](#_bookmark49)). Recognizing this, the SYRCLE risk-of-bias tool for experimental animal studies asks whether groups were “similar at baseline or were they adjusted for confounders in the analysis”([Hooijmans *et al.* 2014](#_bookmark19)). In the context of an animal study, this element would include consideration of covariates such as body weight, litter size, or other outcome- specific covariates. Similarly, the 2012 risk-of-bias guidance from AHRQ recommends consideration of confounding for randomized clinical trials. For this tool, we have only included the consideration of confounding in controlled exposure studies (i.e., experimental human or animal studies) under “other potential threats to internal validity” for cases where it is strongly suspected because randomization and allocation concealment should address the issue of confounding. The issue of confounding overlaps with randomization and allocation concealment, and multiple questions would address the same issue.

## From Cochrane Handbook

## In parallel group experimental studies, sample size is usually limited due to logistics involving exposure set-up. Thus, there is considerably concerns for differences between the exposure groups, which persists after randomization. Thus for parallel group designs, it needs to be checked whether evidence for absence of confounding is provided.

## Crossover studies minimize confounding from personal characteristics and behavior because of the within subject comparison. However, confounding may occur from the choice of order of the exposure conditions. Day of week or time of day may be a relevant factor for the selected outcome and it thus needs to be checked whether the design and conduct of the study accounted for this. Also other external factors (e.g. temperature) may be relevant for symptom reporting. Another critical issue is potential carry-over effects, which biases to the null effect. It needs to be checked whether time between exposure conditions was long enough to avoid carry-over effects.

**Unintended co-exposures for experimental studies if various exposure conditions are delivered at different places**: Evidence of other exposures that are anticipated to bias results should be noted as direct (definitely high risk of bias) or indirect (probably high risk of bias) evidence of other exposures anticipated to bias results, if present and not appropriately adjusted for. Non-differential co-exposures that are likely to bias the results toward the null should be considered in the context of the study findings.

1. Human controlled trial study design used here refers to studies in humans with a controlled exposure including randomized controlled trials and non-randomized experimental studies. [↑](#footnote-ref-1)
2. Note purity thresholds should be developed for specific research questions and reflect empirical data for the substance and outcome under consideration when possible. Therefore, the appropriate cut-off purity value may be lower or higher than the values listed below for ≥99% defining the difference between “definitely low” and “probably low” or ≥98% defining the difference between “probably low” and “probably high” risk of bias. [↑](#footnote-ref-2)
