# Supplementary material for: The effects of radiofrequency electromagnetic fields exposure on human self-reported symptoms: A protocol for a systematic review of human experimental studies
Source: Environ Int. 2022 Jan;158:106953. doi: 10.1016/j.envint.2021.106953 (PMC8668868; doi:10.1016/j.envint.2021.106953)
Supplement: Supplementary data 4 [file mmc4.docx]

**Title of submitted paper and corresponding author**: The effects of radiofrequency electromagnetic fields exposure on human self-reported symptoms: a protocol for a systematic review of human experimental studies – Xavier Bosch-Capblanch

| **#** | **Item** | **Guidance** | **On page #** | **Author Comments** |
| --- | --- | --- | --- | --- |
| **Title** | | | | |
| 1 | Identification | Identify the report as a systematic review, or systematic review and meta-analysis, as appropriate. | 1 | The effects of radiofrequency electromagnetic fields exposure on human self-reported symptoms: a protocol for a systematic review of human experimental studies |
| 2 | Update | If the protocol is for an update of a previous systematic review, identify as such. | Not applicable | - |
| **Registration** | | | | |
| 3 | Registration | If registered, provide the name of the registry (e.g., PROSPERO) and registration number. | Not applicable | Submitted to PROSPERO. Awaiting registration. |
| **Authors** | | | | |
| 4 | Contact | Provide name, institutional affiliation, and e-mail address of all protocol authors; provide physical mailing address of corresponding author. | 1 | Swiss Tropical and Public Health Institute, Socinstrasse 57, CH-4051 Basel, Switzerland  University of Basel, Petersplatz 1, CH-4003 Basel, Switzerland |
| 5 | Contributions | Describe contributions of protocol authors and identify the guarantor of the review. | 17 | MR has led the review team; Jos Verbeek and Gunnhild Oftedal drafted the first version of the protocol. ME and XBC have drafted and finalized the manuscript. SD and HJ have piloted the review instruments. JE has developed and implemented the literature search strategy. All authors have commented the various draft versions of the manuscripts. |
| **Amendments** | | | | |
| 6 | Amendments | If the protocol represents an amendment of a previously completed or published protocol, identify as such and list changes; otherwise, state plan for documenting important protocol amendments. | Not applicable |  |
| **Support** | | | | |
| 7 | Sources | Indicate sources of financial or other support for the review. | 17 | This project is funded by the World Health Organization |
| 8 | Sponsor | Provide name for the review funder/s and/or sponsor/s | Not applicable |  |
| 9 | Roles | Describe roles of funder(s), sponsor(s), and/or institution(s), if any, in developing the protocol. | 17 | WHO officials have commented on the content of the protocol, particularly on the complementarity of the different reviews commissioned. |
| **Introduction** | | | | |
| 10 | Rationale | Describe the rationale for the review in the context of what is already known | 5 | Also 3 and 4. |
| 11 | Objectives | Provide an explicit statement of the question(s) the review will address, with specific reference to:   - Participants - Interventions / Exposures (as appropriate) - Comparisons - Outcomes - Study design | 5 and 6 | in volunteers with IEI-EMF and without IEI-EMF (P), is exposure to RF-EMF (E), as compared to no or lower exposure levels (C), related to immediate effects on symptoms (O)? |
| **Methods** | | | | |
| 12 | Eligibility criteria | Specify the study characteristics (e.g., PICO/PECO, study design, setting, time frame) and report characteristics (e.g., years considered, language, publication status) to be used as criteria for eligibility for the review. | 6 to 9 | We will include trials with RF-EMF exposure, conducted either in laboratories or at any other locations, such as in homes or workplaces:  • randomized trials comparing at least two arms exposed to different intensities of EMF (i.e. parallel group trials);  • randomised crossover trials in which each participant receives all exposure (or alternative exposure) conditions and is randomly allocated to the sequence of those conditions (i.e. crossover trial. |
| 13 | Information sources | Describe all intended information sources (e.g., electronic databases, contact with study authors, trial registers, or other grey literature sources) with planned dates of coverage. | 9 | Medline, Web of Science, PsycInfo, Cochrane Library, Epistemonikos and Embase |
| 14 | Search strategy | Present draft of search strategy to be used for at least one electronic database, including planned limits, such that it could be repeated. | - | See appendix 1. |
| 15 | Data management | Describe the mechanism(s) that will be used to manage records and data throughout the review. | 9 to 11 | After the retrieval of references, we will identify and discard duplicates. First, the relevance of identified papers will be checked on the basis of titles and abstracts, to discard animal studies or studies obviously out of the scope of this review. |
| 16 | Selection process | State the process that will be used for selecting studies (e.g., two independent reviewers) through each phase of the review (i.e., screening, eligibility, and inclusion in meta-analysis). | 9 to 11 | First, the relevance of identified papers will be checked on the basis of titles and abstracts, to discard animal studies or studies obviously out of the scope of this review. The full text of relevant references will be obtained to assess whether they fulfil all the inclusion criteria, independently by the two reviewers. |
| 17 | Data collection process | Describe planned method of extracting data from reports (e.g., piloting forms, done independently, in duplicate), any processes for obtaining and confirming data from investigators. | 10 and 11 | Two reviewers will independently extract and record the relevant data of each eligible study. Discrepancies will be solved by checking the source. If disagreement occurs between the reviewers, this will be resolved by discussion; if no consensus can be reached a third reviewer will be involved. |
| 18 | Data items | List and define all variables for which data will be sought (e.g., PICO items, funding sources), any pre-planned data assumptions and simplifications | 10 and 11 | - Citation  - Study design  - Type of environment (e.g. laboratory, home)  - Participants information (e.g. age, gender, IEI-EMF status -with types of RF-EMF exposure that are experienced to cause the symptoms if applicable, education level, socio-economic and health conditions)  - Number of participants with and without IEI-EMF, enrolled and analysed  - Exposure details (e.g. source, frequency, modulation and duty circle, time pattern of exposure (on-off periods for intermittent exposure), part of body exposed, distance to source)  - Time-average SAR(s), time-average exposure level(s) (incident electric/magnetic field strength or incident power density) or, if exposure levels are not provided, time-average output power(s)  … |
| 19 | Outcomes and prioritisation | List and define all outcomes for which data will be sought, including prioritization of main and additional outcomes, with rationale. | 9 | Symptoms  - For dichotomous outcomes (i.e. symptom yes/no) we will report rate ratios based on the number of persons that report one or more symptom in the intervention and control arms...  Perception  - For dichotomous ratings (presence/absence of field), we will extract the number of correct ratings, as well as false-positive and false-negative recordings during or up to 24 hours after exposure. |
| 20 | Bias in individual studies | Describe anticipated methods for assessing risk of bias of individual studies, including whether this will be done at the outcome or study level, or both; state how this information will be used in data synthesis | 11 and 12 | For evaluating the internal validity, we will conduct a ROB assessment using the "ROB Rating Tool for Human and Animal Studies" |
| 21 | Data synthesis criteria | Describe criteria under which study data will be quantitatively synthesized | 12 to 14 | We will combine the effects of exposure from studies that are considered clinically and statistically sufficiently similar (see section on heterogeneity 3.6.1) in a random effects meta-analysis with STATA version SE 15. |
| 22 | Summary measures | If data are appropriate for quantitative synthesis, describe planned summary measures, methods of handling data, and methods of combining data from studies, including any planned exploration of consistency (e.g., I^2^, Kendall’s tau). | 14 | We will use Stata to assess statistical heterogeneity by means of the I2 statistic. We will take the values of I2 of 25%, 50% and 75% as low, moderate and high degrees of heterogeneity respectively. We will also calculate Tau-square and calculate an 80% prediction interval. |
| 23 | Additional analyses | Describe any proposed additional analyses (e.g., sensitivity or subgroup analyses, meta-regression) | 12 and 14 | To assess relations between exposure doses and the effects on symptoms, we will carry out meta-regression analyses, where exposure will be regarded as a continuous variable. |
| 24 | Alternative synthesis | If quantitative synthesis is not appropriate, describe the type of summary planned | Not applicable |  |
| 25 | Meta-bias | Specify any planned assessment of meta-bias(es) (e.g., publication bias across studies, selective reporting within studies) | 16 | where enough studies are available per outcome (n ≥ 10), we will test publication bias based on standard meta-analytic tests (e.g. Egger's test) |
| 26 | Confidence in cumulative evidence | Describe how the strength of the body of evidence will be assessed (e.g., GRADE) | 14 and 15 | We will use an elaborated GRADE approach (Morgan 2016) to assess the confidence in the evidence for exposure-outcome combinations |

*Environment International* modified PRISMA-P report adapted from: Moher D, Shamseer L, Clarke M, Ghersi D, Liberati A, Petticrew M, Shekelle P, Stewart LA. Preferred Reporting Items for Systematic Review and Meta-Analysis Protocols (PRISMA-P) 2015 statement. Syst Rev. 2015;4(1):1. (Changes are minor, with text edits to accommodate the subject matter of the journal and formatting to fit page.)
